# Streptococcus agalactiae *npx* Is Required for Survival in Human Placental Macrophages and Full Virulence in a Model of Ascending Vaginal Infection during Pregnancy

**DOI:** 10.1128/mbio.02870-22

**Published:** 2022-11-21

**Authors:** Jacky Lu, Rebecca E. Moore, Sabrina K. Spicer, Ryan S. Doster, Miriam A. Guevara, Jamisha D. Francis, Kristen N. Noble, Lisa M. Rogers, Julie A. Talbert, Michelle L. Korir, Steven D. Townsend, David M. Aronoff, Shannon D. Manning, Jennifer A. Gaddy

**Affiliations:** a Department of Pathology, Microbiology and Immunology, Vanderbilt University grid.152326.1grid.412807.8 Medical Center, Nashville, Tennessee, USA; b Department of Chemistry, Vanderbilt University grid.152326.1grid.412807.8 , Nashville, Tennessee, USA; c Department of Medicine, Vanderbilt University grid.152326.1grid.412807.8 Medical Center, Nashville, Tennessee, USA; d Department of Medicine, University of Louisville, Louisville, Kentucky, USA; e Department of Pediatrics, Vanderbilt University grid.152326.1grid.412807.8 Medical Center, Nashville, Tennessee, USA; f Department of Medicine, Indiana University, Indianapolis, Indiana, USA; g Department of Microbiology and Molecular Genetics, Michigan State University, East Lansing, Michigan, USA; h Department of Biology, Aurora University, Aurora, Illinois, USA; i Tennessee Valley Healthcare Systems, Department of Veterans Affairs, Nashville, Tennessee, USA; j Center for Medicine Health and Society, Vanderbilt University grid.152326.1grid.412807.8 , Nashville, Tennessee, USA; Louis Stokes Veterans Affairs Medical Center

**Keywords:** *Streptococcus*, infection, innate immunity, reactive oxygen species, ROS

## Abstract

Streptococcus agalactiae, also known as group B Streptococcus (GBS), is a Gram-positive encapsulated bacterium that colonizes the gastrointestinal tract of 30 to 50% of humans. GBS causes invasive infection during pregnancy that can lead to chorioamnionitis, funisitis, preterm prelabor rupture of membranes (PPROM), preterm birth, neonatal sepsis, and maternal and fetal demise. Upon infecting the host, GBS encounters sentinel innate immune cells, such as macrophages, within reproductive tissues. Once phagocytosed by macrophages, GBS upregulates the expression of the gene *npx*, which encodes an NADH peroxidase. GBS mutants with an *npx* deletion (Δ*npx*) are exquisitely sensitive to reactive oxygen stress. Furthermore, we have shown that *npx* is required for GBS survival in both THP-1 and placental macrophages. In an *in vivo* murine model of ascending GBS vaginal infection during pregnancy, *npx* is required for invading reproductive tissues and is critical for inducing disease progression, including PPROM and preterm birth. Reproductive tissue cytokine production was also significantly diminished in Δ*npx* mutant-infected animals compared to that in animals infected with wild-type (WT) GBS. Complementation in *trans* reversed this phenotype, indicating that *npx* is critical for GBS survival and the initiation of proinflammatory signaling in the gravid host.

## INTRODUCTION

Preterm deliveries occur at less than 37 weeks of gestation (1). Despite advancing knowledge of risk factors and the introduction of public health and medical interventions to reduce the occurrence, the rate of preterm birth (PTB) in the United States and other developed countries still hovers at between 5 and 9% (2, 3). Preterm births account for 75% of perinatal mortality and over 50% of long-term morbidity (4). While most preterm babies survive, these infants are at an increased risk of neurodevelopmental impairments, respiratory conditions, and gastrointestinal complications (5). Intrauterine infection may account for at least 25 to 40% of preterm births (6).

Streptococcus agalactiae, or group B Streptococcus (GBS), is an encapsulated Gram-positive bacterium that colonizes the urogenital tract and lower gastrointestinal tract of 30% of healthy adults (7). Although GBS is a common member of the intestinal microbiota, it can cause invasive infections during pregnancy, leading to sepsis or meningitis in the neonate (8). Indeed, GBS is a leading cause of adverse pregnancy and neonatal outcomes such as stillbirth, chorioamnionitis, preterm birth, and neonatal sepsis (9); up to 25% of invasive GBS infections during pregnancy end in stillbirth or spontaneous abortion (10). To prevent neonatal infection, the Centers for Disease Control and Prevention (CDC) recommends screening mothers for GBS late in the third trimester and administering antibiotic therapy to those who test positive during labor (11). There is a concern, however, that antibiotic exposure could alter the infant’s developing microbiome, which may contribute to lifelong consequences (8), and intrapartum antibiotic prophylaxis does not prevent late-onset disease, stillbirth, or preterm birth (12). Consequently, GBS remains the leading infectious cause of morbidity and mortality among neonates in the United States (13).

GBS pathogenesis begins with adherence to vaginal epithelial cells (8). For successful colonization, the bacteria can form biofilm structures to evade the immune system (14). Following vaginal colonization, GBS can ascend above the cervical os, through as-yet-undefined mechanisms, and traverse the fetal membranes, causing fetal infection (15). The inflammation of extraplacental (“fetal”) membranes in response to GBS infection is termed chorioamnionitis (16).

Because the human immune system has evolved to protect against pathogens, this paradigm is more complex during pregnancy as the system must defend the gravid uterus against infection and maintain immunologic tolerance to the semiallogeneic fetus. This careful balance must be maintained to prevent harm to the mother and fetus. Consequently, the immune response during pregnancy is characterized by dynamic modifications of the maternal and fetal tissues reliant on the stage of pregnancy (17, 18). Bacteria commonly cause intrauterine infections, triggering a proinflammatory response originating in the decidua by the activation of pattern recognition receptors (PRRs), which may result in preterm birth (19, 20). Previous mouse studies have demonstrated that innate immune signaling is sufficient to instigate adverse pregnancy outcomes (18). The presence of proinflammatory cytokines, including interleukin-1β (IL-1β), IL-6, IL-8, and tumor necrosis factor alpha (TNF-α), in the amnionic fluid or cervicovaginal lavage fluid of patients is indicative of the onset of preterm labor (21–23). A variety of leukocytes responsible for cytokine production are present in the reproductive tissues, including maternal natural killer cells, dendritic cells, macrophages, and lymphocytes (24). In particular, macrophages represent a predominant subset of human leukocytes that serve as antigen-presenting cells (APCs) in the decidua, comprising 20 to 25% of all decidual leukocytes (25).

Placental macrophages (PMs) represent a mixed population of maternally and fetally derived cells (26) that are thought to play critical roles in placental invasion, angiogenesis, tissue modeling, and development (27, 28). Recently, studies have demonstrated that PMs defend against invading bacterial pathogens by the release of macrophage extracellular traps (29).

Upon phagocytosis by a macrophage, bacterial pathogens are trapped in a phagosome, which is a highly oxidative environment (30). Additionally, cells generate reactive oxygen species (ROS) as metabolic by-products. The level of ROS produced by the NOX2 NADPH oxidase in macrophages is significantly higher under conditions of infection than in resting states; hence, stimulating oxidative stress to kill invading pathogens is a critical pathway for innate immunity (30). ROS can damage macromolecules, including lipids, proteins, and nucleic acids, ultimately leading to cell death (31). Bacterial pathogens have evolved strategies to survive in highly oxidative environments, such as by producing antioxidants or enzymes that can inactivate and detoxify ROS (32). GBS produces several products to help circumnavigate ROS stress during infection, including superoxide dismutase, which converts superoxide to H_2_O_2_ and O_2_ (33). GBS also produces glutathione and a carotenoid pigment, which protect against oxidative damage (34, 35). A previous study revealed that GBS *npx* encodes an NADH peroxidase that is critical for the detoxification of and resistance to peroxide stress and survival within THP-1 macrophage-like cells (36). The deletion of the *npx* locus in a Δ*npx* mutant resulted in an attenuated ability to detoxify peroxide, a result that was reversed via genetic complementation in *trans* (36).

During ascending vaginal infections in pregnancy, some of the first immune cells that GBS encounters at the maternal-fetal interface are placental macrophages. We hypothesized that *npx* is also required for GBS to survive within PMs and to cause disease progression during pregnancy. To test this, we sought to characterize its role in pathogenesis *in vivo* using an established pregnant mouse model of ascending vaginal infection and *ex vivo* using primary human PMs. Here, we demonstrate that *npx* is required for GBS survival in PMs, full virulence, and invasion of gravid reproductive tissues *in vivo*. We have also demonstrated that *npx* is required for the induction of specific inflammatory cytokines expressed during GBS infection.

## RESULTS

### Transmission electron microscopy visualization of intracellular GBS in placental macrophages.

**FIG 1:**
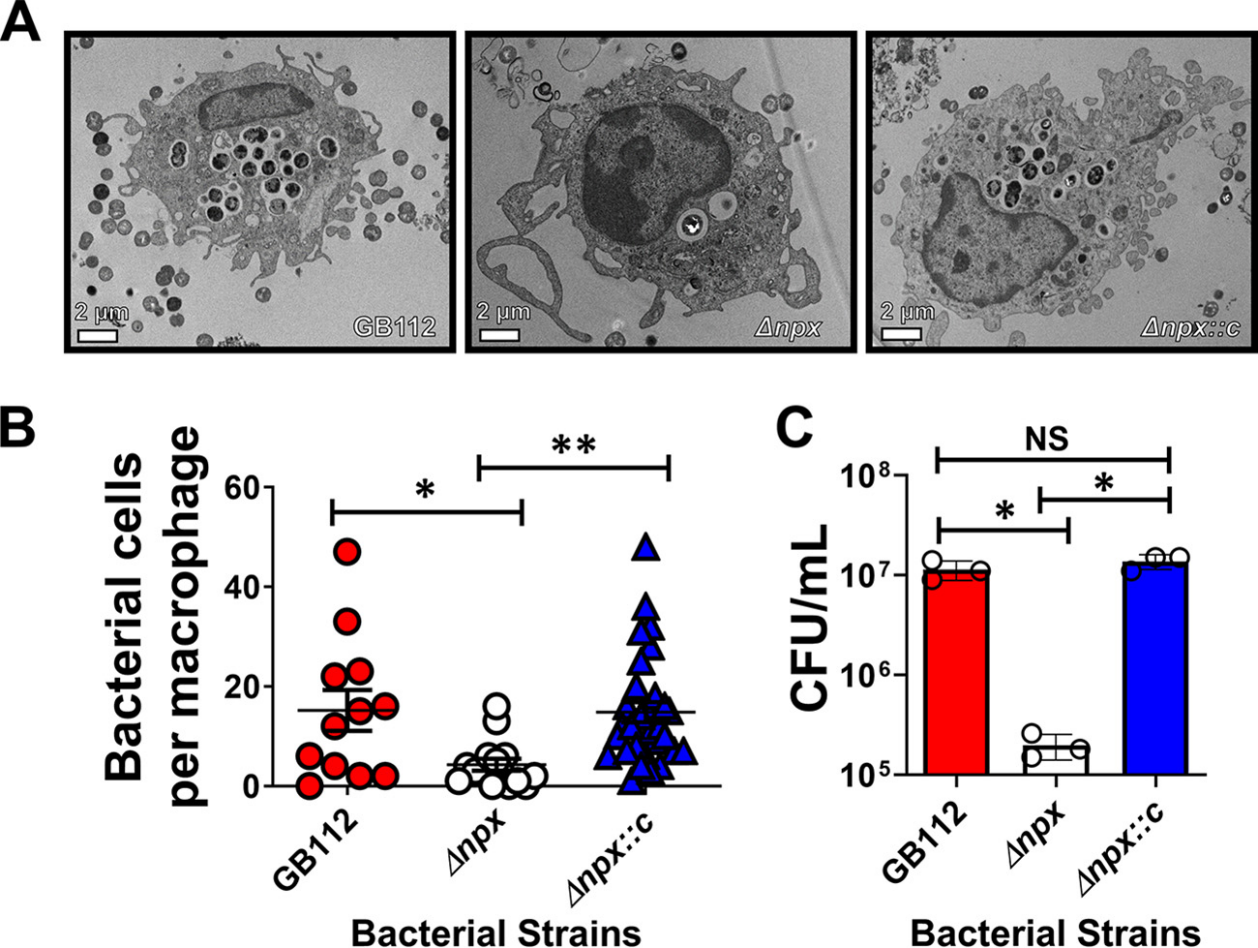
Analysis of GBS survival in primary human placental macrophage cells. (A) Transmission electron microscopy analyses reveal that *npx* is required for GBS survival in placental macrophages. (B) Enumeration of bacterial cells per placental macrophage via electron microscopy analyses indicates that WT GBS (GB112) (red) survives within primary human placental macrophages, but an isogenic Δ*npx* mutant (white) is attenuated in intracellular survival compared to both the parental strain (GB112) and the complemented derivative (Δ*npx::c*) (blue). (C) Quantitative culture analysis of bacterial survival by gentamicin protection assays reveals that *npx* is required for GBS survival in placental macrophages. *, *P* < 0.05; **, *P* < 0.01 (by one-way ANOVA with Tukey’s *post hoc* multiple-comparison test). NS, statistically indistinguishable (3 biological replicates).

Transmission electron microscopy (TEM) was used to visualize intracellular GB112 and isogenic mutants in PMs. This analysis revealed that few intracellular Δ*npx* cells were observed compared to wild-type (WT) GB112 and an isogenic complemented Δ*npx* derivative harboring a plasmid containing the *npx* locus (Δ*npx::c*) (Fig. 1A). Indeed, PM samples infected with WT GB112 averaged 17 bacterial cells per macrophage, while PMs infected with the Δ*npx* mutant averaged 5 bacterial cells per macrophage. This result was reversed in the strain with a genetic complementation of the *npx* allele in *trans*, which infected an average of 16 bacterial cells per macrophage (Fig. 1B). Quantitative culture analyses of living bacterial cells in PMs via gentamicin protection assays demonstrated that an average of 1.1 × 10^7^ WT GBS cells survived per 5 × 10^5^ to 1 × 10^6^ macrophages, compared to an average of 2.0 × 10^5^ cells for the Δ*npx* mutant (*P* < 0.05 by one-way analysis of variance [ANOVA] with Tukey’s *post hoc* test) (Fig. 1C). Meanwhile, complementation with the Δ*npx::c* vector restored survival inside macrophages, with an average of 1.4 × 10^7^ cells surviving per 5 × 10^5^ to 1 × 10^6^ macrophages, a result that was comparable to those for WT GB112 (*P* < 0.01 by one-way ANOVA with Tukey’s *post hoc* test). Taken together, these results show that *npx* plays an important role in persistence and survival within PMs.

### Placental macrophage cytokine responses to intracellular GBS infection.

**FIG 2:**
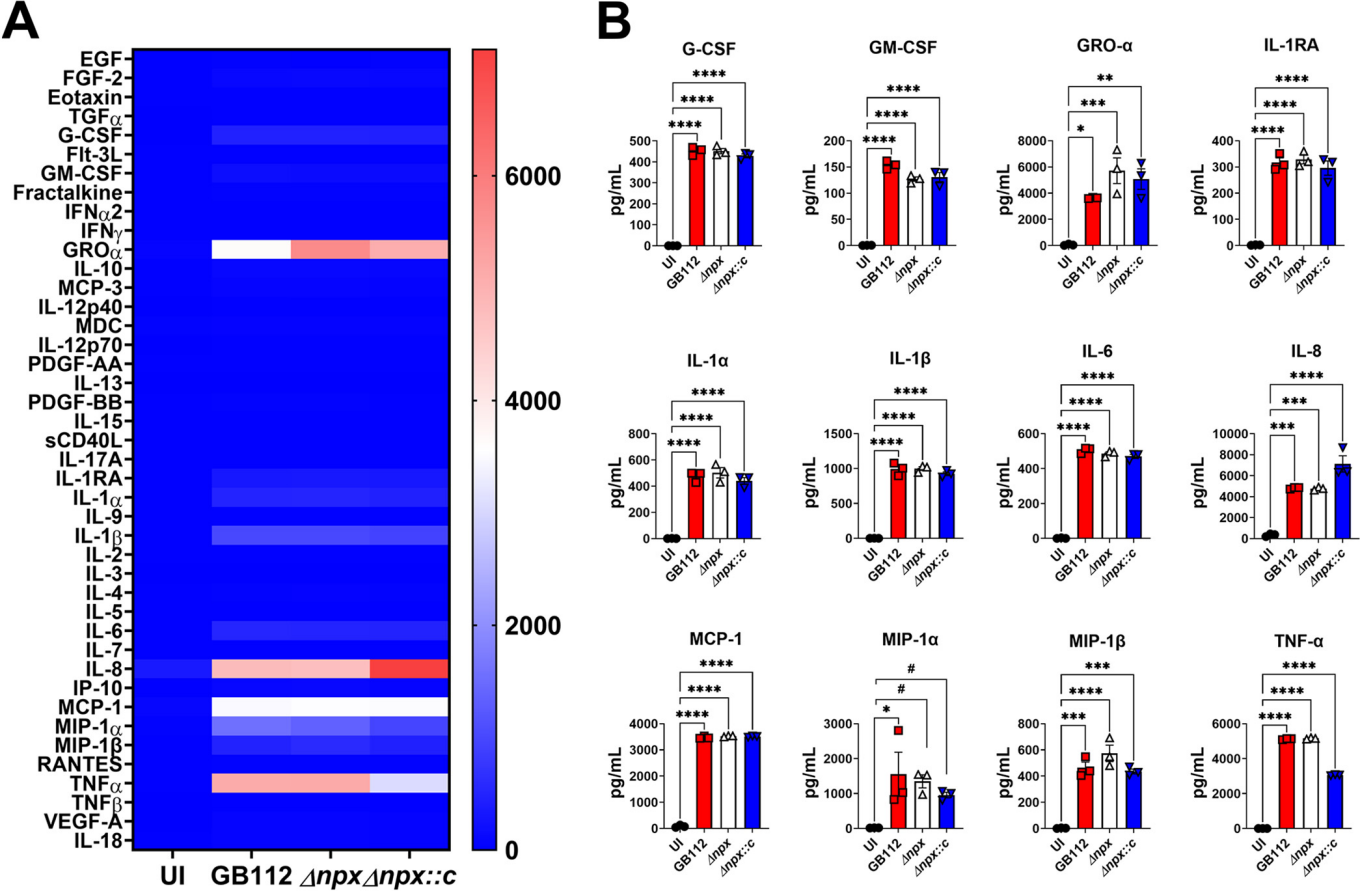
GBS *npx* expression is dispensable for the initiation of proinflammatory cytokine production by human placental macrophages. (A) Heat map results (blue, low levels; red, high levels) of multiplex cytokine analyses of primary human placental macrophage-secreted fractions after coculture with wild-type GB112, the isogenic Δ*npx* mutant, or the isogenic complemented Δ*npx* derivative (Δ*npx::c*) reveal that GBS infection induces the production of multiple proinflammatory cytokines compared to the uninfected controls (UI). EGF, epidermal growth factor; FGF, fibroblast growth factor; TGFα, transforming growth factor α; IFNα2, interferon alpha 2; PDGF-AA, platelet-derived growth factor AA; sCD40L, soluble CD40 ligand; VEGF-A, vascular endothelial growth factor A. (B) Quantitation of cytokine levels reveals the significantly enhanced production of G-CSF, GM-CSF, GRO-α, IL-1RA, IL-1α, IL-1β, IL-6, IL-8, MCP-1, MIP-1α, MIP-1β, and TNF-α in macrophages cocultured with the GB112 (red), Δ*npx* (white), and Δ*npx::c* (blue) strains compared to the uninfected controls (black). *, *P* < 0.05; **, *P* < 0.01; ***, *P* < 0.001; ****, *P* < 0.0001 (by one-way ANOVA with Tukey’s *post hoc* multiple-comparison test). #, *P* < 0.05 (by Student’s *t* test with Welch’s correction) (3 biological replicates).

Because we observed a difference in intracellular survival among the GB112 (WT), Δ*npx*, and Δ*npx::c* strains, we hypothesized that there may be differences in cytokine production by PMs. Following infection of PMs with GBS and its isogenic mutants overnight, the cell supernatants were collected, and cytokine quantifications were performed. Intracellular infection with WT GB112 resulted in the increased secretion of a variety of proinflammatory cytokines, including CXC motif ligand 1 (CXCL1), interleukin 1 receptor antagonist (IL-1RA), interleukin 1α (IL-1α), interleukin 1β (IL-1β), interleukin 6 (IL-6), interleukin 8 (IL-8), monocyte chemoattractant protein 1 (MCP-1), macrophage inflammatory protein 1α (MIP-1α), macrophage inflammatory protein 1β (MIP-1β), and tumor necrosis factor alpha (TNF-α) (Fig. 2). Nonetheless, these cytokines were similarly increased in PMs infected with the Δ*npx* and Δ*npx::c* strains, suggesting that *npx* does not influence the production of proinflammatory cytokines by PMs *ex vivo*.

### GBS *npx* is associated with preterm birth in a mouse model of ascending infection.

**FIG 3:**
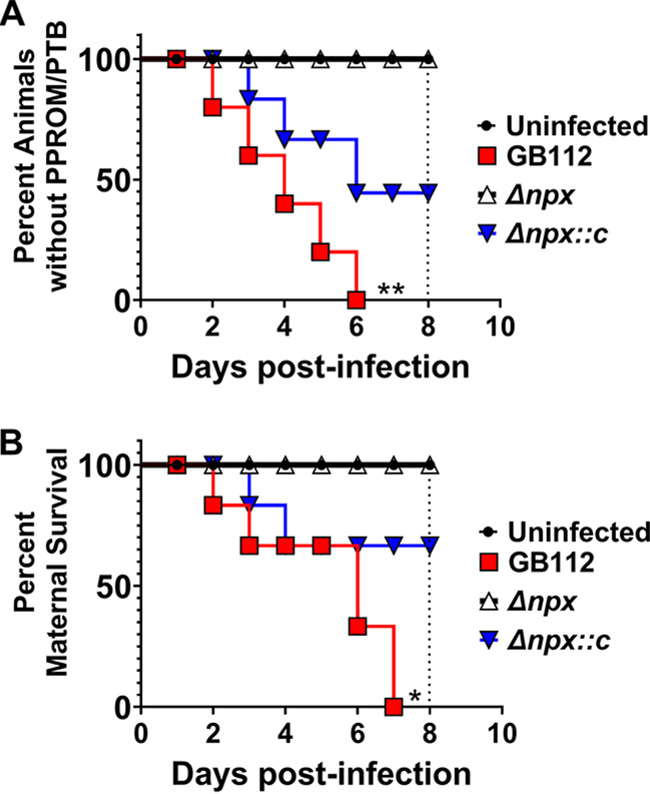
Analysis of the role of GBS *npx* in disease progression in a mouse model of ascending vaginal infection during pregnancy. (A) Evaluation of the incidence of (percentage of animals exhibiting) preterm premature rupture of membranes (PPROM) and preterm birth (PTB) over time (8 days postinfection and term for gestation). Wild-type GB112-infected animals (red) had higher incidences of PPROM and PTB than did uninfected (black) and Δ*npx* mutant-infected (white) animals, with 100% of the GB112-infected cohort experiencing PPROM or PTB by 6 days postinfection. Complementation of the *npx* locus in *trans* (Δ*npx::c*) (blue) resulted in 65% PTB and PPROM by 6 days postinfection. (B) Evaluation of maternal mortality over time (8 days postinfection and term for gestation). GB112-infected animals had a 100% maternal mortality rate by 7 days postinfection, a rate that was significantly higher than those for the uninfected control and Δ*npx* mutant-infected cohorts, which exhibited 0% maternal mortality by 8 days postinfection. Complementation in *trans* partially restored mortality (33% maternal death by 8 days postinfection) (*n* = 6 to 9 dams total from 3 separate experiments) (*, *P* < 0.05; **, *P* < 0.01 [by a Mantel-Cox log rank test and a Gehan-Breslow-Wilcoxon test]).

To assess the role of GBS *npx in vivo* in the context of ascending infection during pregnancy, we utilized a pregnant mouse model initiated by intravaginal GBS infection (37). Previous work demonstrated that in this model, ascending vaginal GBS infection results in bacterial invasion, inflammation of reproductive tissues, preterm prelabor rupture of membranes (PPROM), preterm birth (PTB), as well as maternal and neonatal demise (37). Hence, we hypothesized that Npx could be important for GBS evasion of innate immune responses in the gravid reproductive tract. Our results (Fig. 3) demonstrate that mice infected with WT GB112 had higher incidences of PPROM and PTB than did uninfected and Δ*npx* mutant-infected animals (*P* = 0.0024 by a Mantel-Cox log rank test and *P* = 0.0067 by a Gehan-Breslow-Wilcoxon test). Notably, all GB112-infected animals experienced PPROM or PTB by 6 days postinfection. Complementation of the *npx* locus in *trans* resulted in 65% PTB and PPROM by 6 days postinfection; these results were statistically indistinguishable from those for cohorts infected with WT GB112 (*P* = 0.0766 by a Mantel-Cox log rank test and *P* = 0.1135 by a Gehan-Breslow-Wilcoxon test). By 7 days postinfection, GB112-infected animals had a 100% maternal mortality rate, which was significantly higher than that for the uninfected controls and Δ*npx* mutant-infected cohorts, which exhibited 0% maternal mortality by 8 days postinfection (*P* = 0.0164 by a Mantel-Cox log rank test and *P* = 0.0442 by a Gehan-Breslow-Wilcoxon test). Complementation in *trans* partially decreased the mortality rate (33% maternal death by 8 days postinfection), which was statistically indistinguishable from that for WT GB112 (*P* = 0.2205 by a Mantel-Cox log rank test and *P* = 0.4190 by a Gehan-Breslow-Wilcoxon test).

### Analysis of bacterial burdens in reproductive tissues.

**FIG 4:**
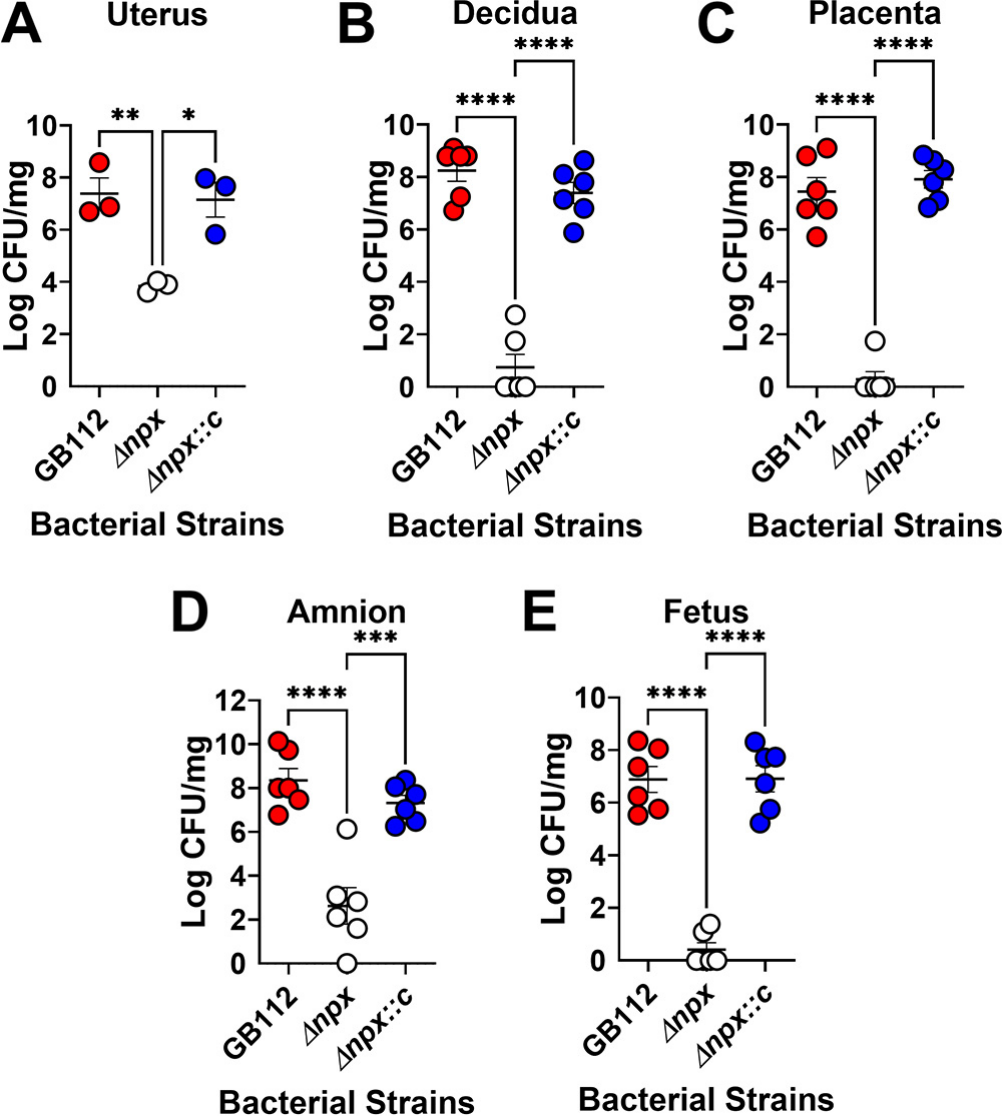
Analysis of bacterial burdens within gravid reproductive tissues. Shown are data from quantitative culture analyses of the bacterial burdens within the uterus (A), decidua (B), placenta (C), amnion (D), and fetus (E) of pregnant mice on embryonic day 15.5, 2 days after infection with either WT GBS (GB112) (red), an isogenic Δ*npx* mutant (white), or the isogenic complemented derivative (Δ*npx::c*) (blue). GBS *npx* is required for full bacterial burdens in reproductive tissues in a model of ascending infection during pregnancy. *, *P* < 0.05; **, *P* < 0.01; ***, *P* < 0.001; ****, *P* < 0.0001 (by one-way ANOVA with Tukey’s *post hoc* multiple-comparison test) (*n* = 3 dams total, with 1 to 2 fetal-placental units analyzed per dam).

To quantify the bacterial burden and classify host responses within reproductive tissues, intravaginal infections were performed using an infectious dose of 5 × 10^2^ to 1 × 10^3^ CFU. Mice were sacrificed at 48 h postinfection to collect reproductive tissues for bacteriological and immunological analyses. The bacterial burden was evaluated by quantitative culture methods for reproductive tissues, including uterine, decidual, placental, amnion, and fetal tissues (Fig. 4). The Δ*npx* mutant exhibited a 3.5-log decrease in the burden in uterine tissue compared to the parental strain (*P* < 0.01 by one-way ANOVA with Tukey’s *post hoc* multiple-comparison test). Genetic complementation in *trans* resulted in a significant increase in the bacterial burden (*P* < 0.05 by one-way ANOVA with Tukey’s *post hoc* multiple-comparison test) compared to the Δ*npx* mutant. The Δ*npx* mutant exhibited a 7.5-log decrease in the burden in decidual tissue compared to the parental strain (*P* < 0.0001 by one-way ANOVA with Tukey’s *post hoc* multiple-comparison test). Genetic complementation in *trans* resulted in a significant increase in the bacterial burden (*P* < 0.0001 by one-way ANOVA with Tukey’s *post hoc* multiple-comparison test) compared to the Δ*npx* mutant. The Δ*npx* mutant exhibited a 7.2-log decrease in the burden in placental tissue compared to the parental strain (*P* < 0.0001 by one-way ANOVA with Tukey’s *post hoc* multiple-comparison test). Genetic complementation in *trans* resulted in a significant increase in the bacterial burden (*P* < 0.0001 by one-way ANOVA with Tukey’s *post hoc* multiple-comparison test) compared to the Δ*npx* mutant. The Δ*npx* mutant exhibited a 5.7-log decrease in the burden in amnion tissue compared to the parental strain (*P* < 0.0001 by one-way ANOVA with Tukey’s *post hoc* multiple-comparison test). Genetic complementation in *trans* resulted in a significant increase in the bacterial burden (*P* < 0.001 by one-way ANOVA with Tukey’s *post hoc* multiple-comparison test) compared to the Δ*npx* mutant. The Δ*npx* mutant exhibited a 6.5-log decrease in the burden in fetal tissue compared to the parental strain (*P* < 0.0001 by one-way ANOVA with Tukey’s *post hoc* multiple-comparison test). Genetic complementation in *trans* resulted in a significant increase in the bacterial burden (*P* < 0.0001 by one-way ANOVA with Tukey’s *post hoc* multiple-comparison test) compared to the Δ*npx* mutant. This *in vivo* study revealed that Npx is required for GBS to achieve full bacterial burdens in reproductive tissues in a model of ascending infection during pregnancy.

### Analysis of bacterial growth in amniotic fluid.

Because the Δ*npx* mutant exhibited attenuated bacterial burdens within reproductive tissues compared to the WT or the complemented derivative, we hypothesized that the Δ*npx* mutant might have an attenuated ability to grow in certain environments such as amniotic fluid. To test this, we utilized human and mouse amniotic fluid and performed an inoculation with WT GBS, the Δ*npx* mutant, or the complemented derivative at a 1:100 dilution. Samples were incubated overnight, and viable bacteria were enumerated via serial dilution and quantitative culture techniques. The results indicate that the Δ*npx* mutant is attenuated in its ability to grow in human amniotic fluid and mouse amniotic fluid (*P* < 0.001 and *P* < 0.0001, respectively, by one-way ANOVA with Tukey’s *post hoc* multiple-comparison test), results that were reversed by genetic complementation techniques (see Fig. S1 in the supplemental material).

10.1128/mbio.02870-22.1 FIG S1 Analysis of bacterial viability in human or mouse amniotic fluid. The wild-type GB112 (WT) (red circles), Δ*npx* (white squares), or Δ*npx::c* (blue triangles) strain was grown overnight in human amniotic fluid (A) or mouse amniotic fluid (B), and cell viability (log_10_ CFU per milliliter) was determined by quantitative culture techniques. The Δ*npx* mutant was attenuated in cell viability in human and mouse amniotic fluid, a result that was reversed by genetic complementation *in trans*. ***, *P* < 0.001; ****, *P* < 0.0001 (by one-way ANOVA with Tukey’s *post hoc* multiple-comparison test). Download FIG S1, TIF file, 0.4 MB. Copyright © 2022 Lu et al. 2022 Lu et al. https://creativecommons.org/licenses/by/4.0/ This content is distributed under the terms of the Creative Commons Attribution 4.0 International license.

### Expression of GBS *npx* alters cytokine responses to GBS infection.

**FIG 5:**
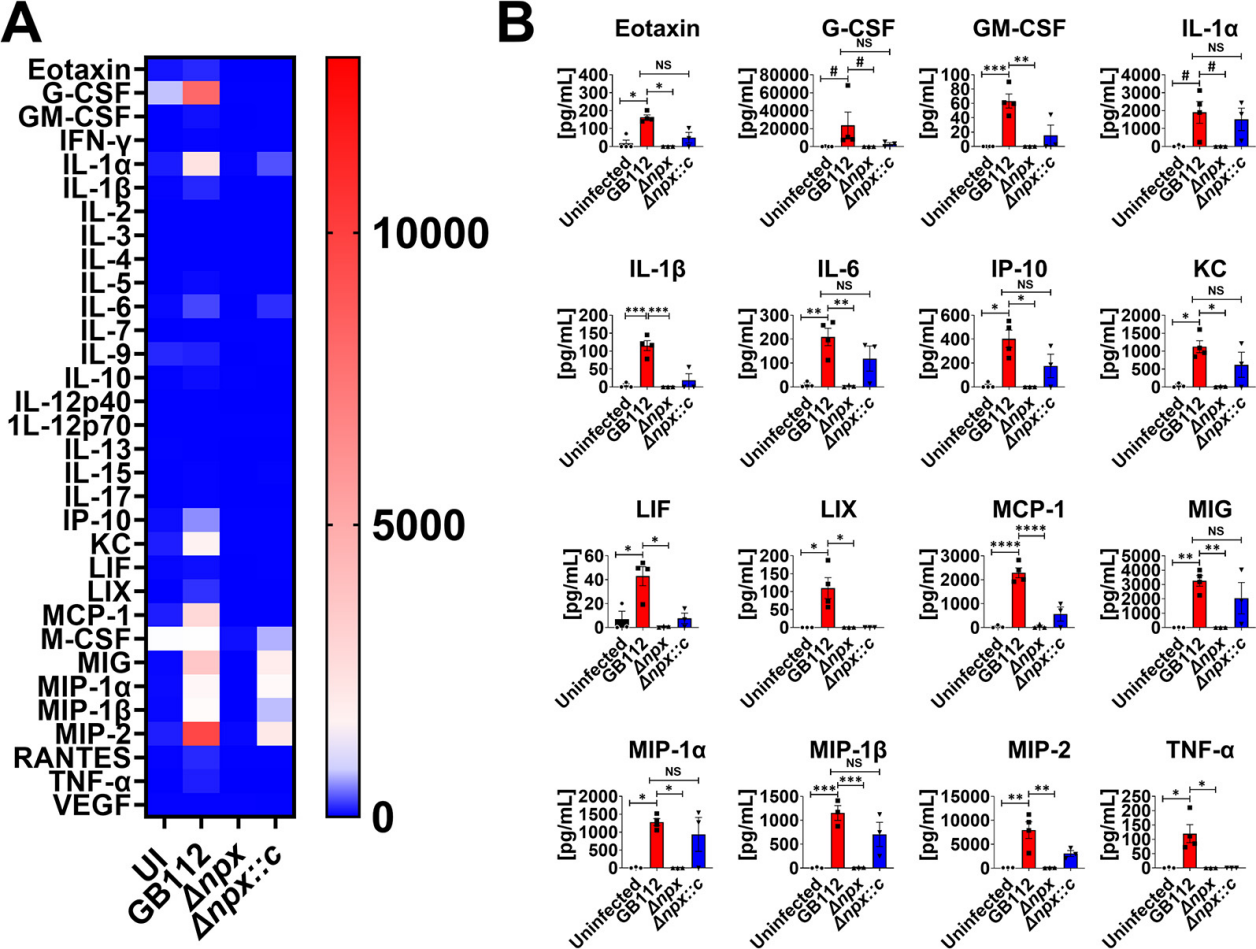
Analysis of cytokine production in uterus tissue in response to GBS infection. (A) Heat map results (blue, low levels; red, high levels) of multiplex cytokine analyses of gestational tissues after ascending vaginal infection with wild-type GB112, the Δ*npx* isogenic mutant, or the isogenic complemented Δ*npx* derivative (Δ*npx::c*) as well as the uninfected controls (UI). Uterine tissues were collected from pregnant mice on embryonic day 15.5, 2 days after vaginal infection with GBS. (B) Quantification of eotaxin, G-CSF, GM-CSF, IL-1α, IL-1β, IL-6, IP-10, KC, LIF, LIX, MCP-1, MIG, MIP-1α, MIP-1β, MIP-2, and TNF-α levels revealed that wild-type GB112 infection (red bars) significantly enhances the production of these cytokines compared to the levels in the uninfected controls (black bars), but the isogenic Δ*npx* mutant (white bars) is significantly attenuated in its ability to induce these cytokines compared to the parental strain. Conversely, the complemented derivative (Δ*npx::c*) (blue bars) is often statistically indistinguishable from the parental strain (not statistically significant [NS]). Bars indicate mean values ± standard errors of the means, with individual data points representing results from uterus tissues from individual dams. #, *P* < 0.05 (by Student’s *t* test). *, *P* < 0.05; **, *P* < 0.01; ***, *P* < 0.001; ****, *P* < 0.0001 (by one-way ANOVA with Tukey’s *post hoc* multiple-comparison test). The results indicate that GBS *npx* is required for the full initiation of proinflammatory cytokine responses in uterine tissues in a model of ascending infection during pregnancy.

**FIG 6:**
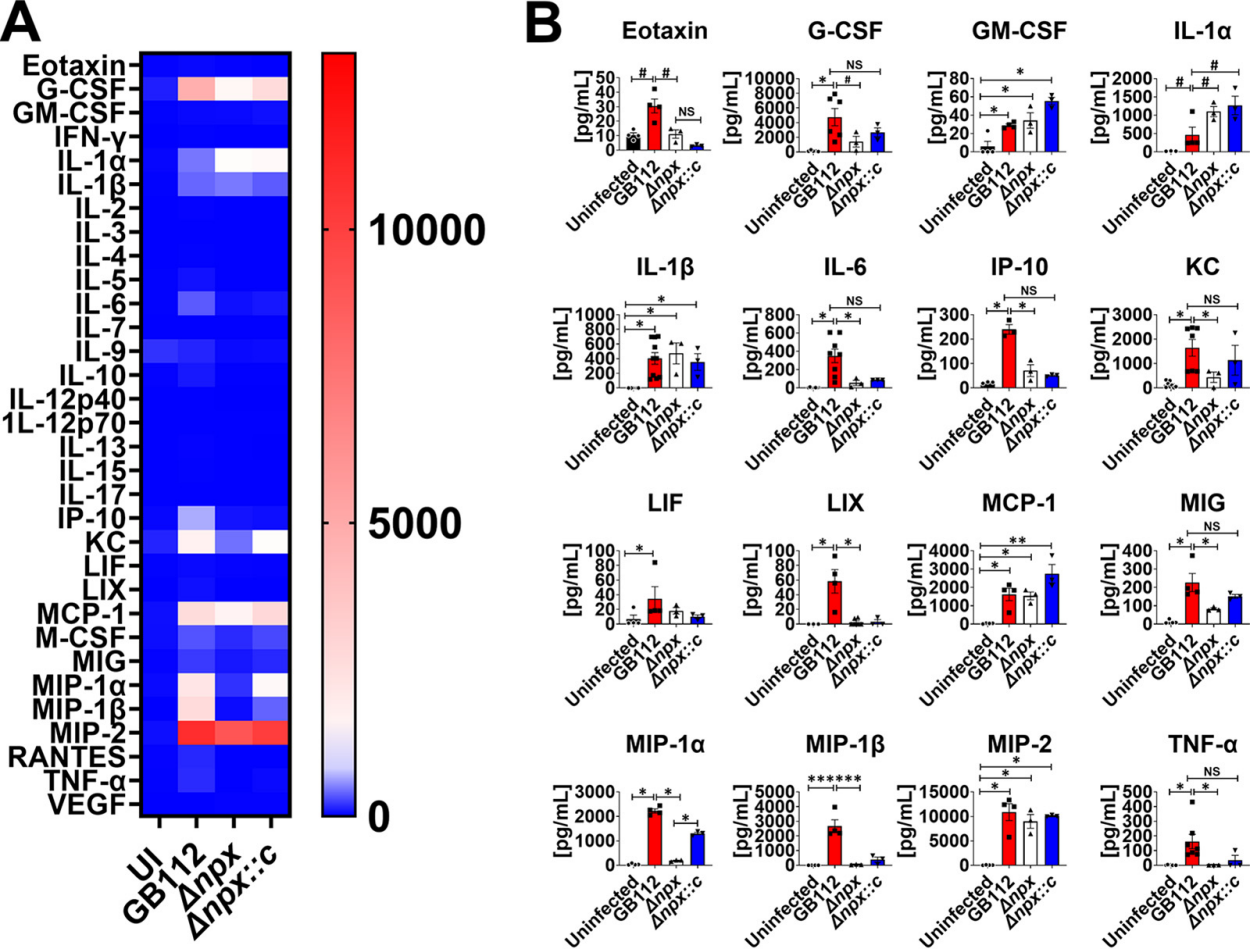
Analysis of cytokine production in decidua tissue in response to GBS infection. (A) Heat map results (blue, low levels; red, high levels) of multiplex cytokine analyses of decidua tissues after ascending vaginal infection with wild-type GB112, the isogenic Δ*npx* mutant (Δ*npx*), or the isogenic complemented Δ*npx* derivative (Δ*npx::c*) as well as the uninfected controls (UI). Decidua tissues were collected from pregnant mice on embryonic day 15.5, 2 days after vaginal infection with GBS. (B) Quantification of eotaxin, G-CSF, GM-CSF, IL-1α, IL-1β, IL-6, IP-10, KC, LIF, LIX, MCP-1, MIG, MIP-1α, MIP-1β, MIP-2, and TNF-α levels revealed that wild-type GB112 infection (red bars) significantly enhances the production of these cytokines compared to the levels in the uninfected controls (black bars), but the isogenic Δ*npx* mutant (white bars) is significantly attenuated in its ability to induce these cytokines compared to the parental strain. Conversely, the complemented derivative (Δ*npx::c*) (blue bars) is often statistically indistinguishable from the parental strain (not statistically significant [NS]). Bars indicate mean values ± standard errors of the means, with individual data points representing results from decidual tissues from individual fetal-placental units from separate dams. #, *P* < 0.05 (by Student’s *t* test). *, *P* < 0.05; **, *P* < 0.01; ***, *P* < 0.001; ****, *P* < 0.0001 (by one-way ANOVA with Tukey’s *post hoc* multiple-comparison test). The results indicate that GBS *npx* is required for the full initiation of proinflammatory cytokine responses in decidual tissues in a model of ascending infection during pregnancy.

**FIG 7:**
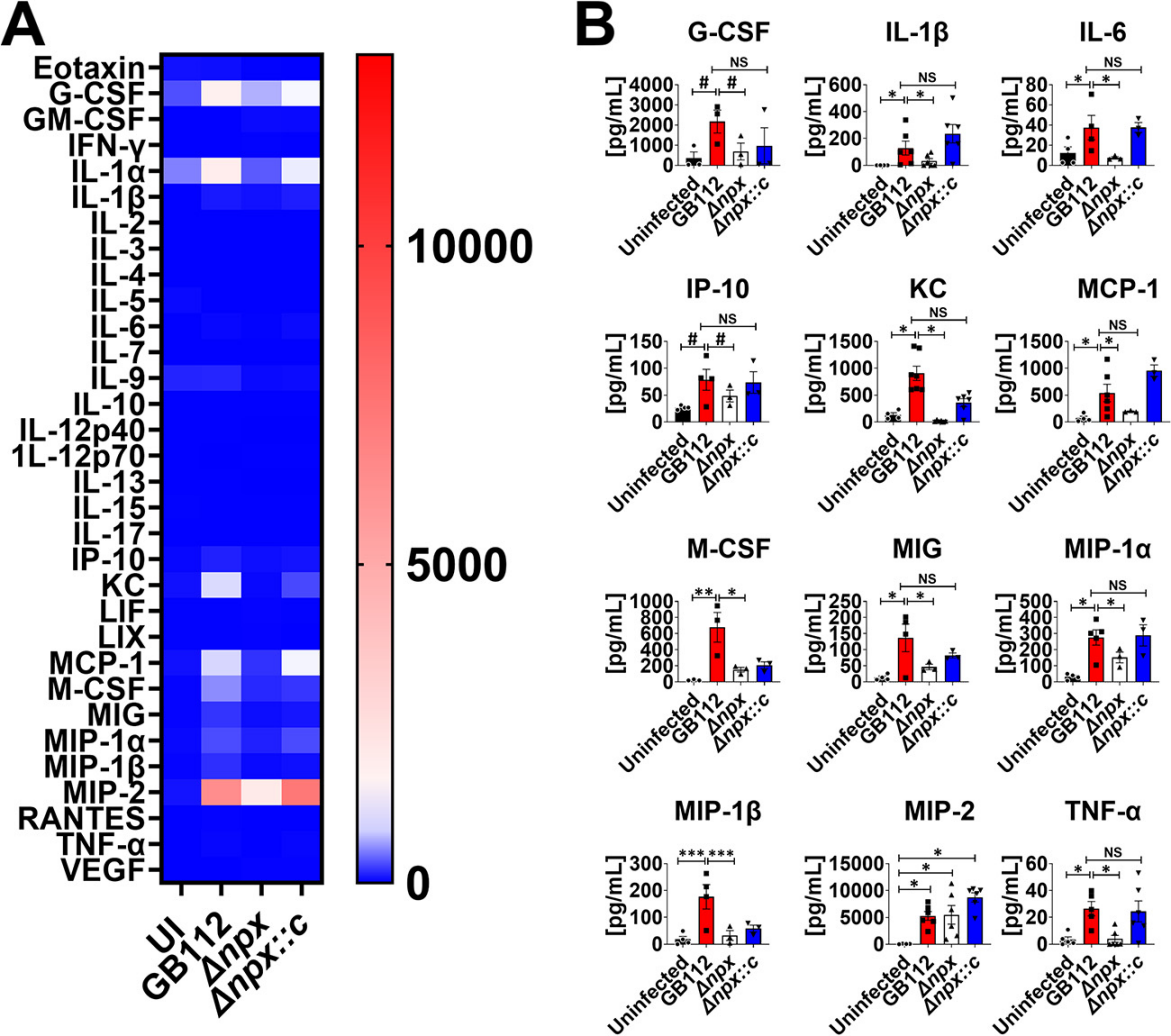
Analysis of cytokine production in placenta tissue in response to GBS infection. (A) Heat map results (blue, low levels; red, high levels) of multiplex cytokine analyses of placenta tissues after ascending vaginal infection with wild-type GB112, the Δ*npx* isogenic mutant, or the complemented isogenic Δ*npx* derivative (Δ*npx::c*) as well as the uninfected controls (UI). Placenta tissues were collected from pregnant mice on embryonic day 15.5, 2 days after vaginal infection with GBS. (B) Quantification of G-CSF, IL-1β, IL-6, IP-10, KC, MCP-1, M-CSF, MIG, MIP-1α, MIP-1β, MIP-2, and TNF-α levels revealed that wild-type GB112 infection (red bars) significantly enhances the production of these cytokines compared to the levels in the uninfected controls (black bars), but the isogenic Δ*npx* mutant (white bars) is significantly attenuated in its ability to induce these cytokines compared to the parental strain, except for MIP-2. Conversely, the complemented derivative (Δ*npx::c*) (blue bars) is often statistically indistinguishable from the parental strain (not statistically significant [NS]). Bars indicate mean values ± standard errors of the means, with individual data points representing results from placental tissues from individual fetal-placental units from separate dams. #, *P* < 0.05 (by Student’s *t* test). *, *P* < 0.05; **, *P* < 0.01; ***, *P* < 0.001; ****, *P* < 0.0001 (by one-way ANOVA with Tukey’s *post hoc* multiple-comparison test). The results indicate that GBS *npx* is required for the initiation of numerous proinflammatory cytokine responses in placental tissues in a model of ascending infection during pregnancy.

**FIG 8:**
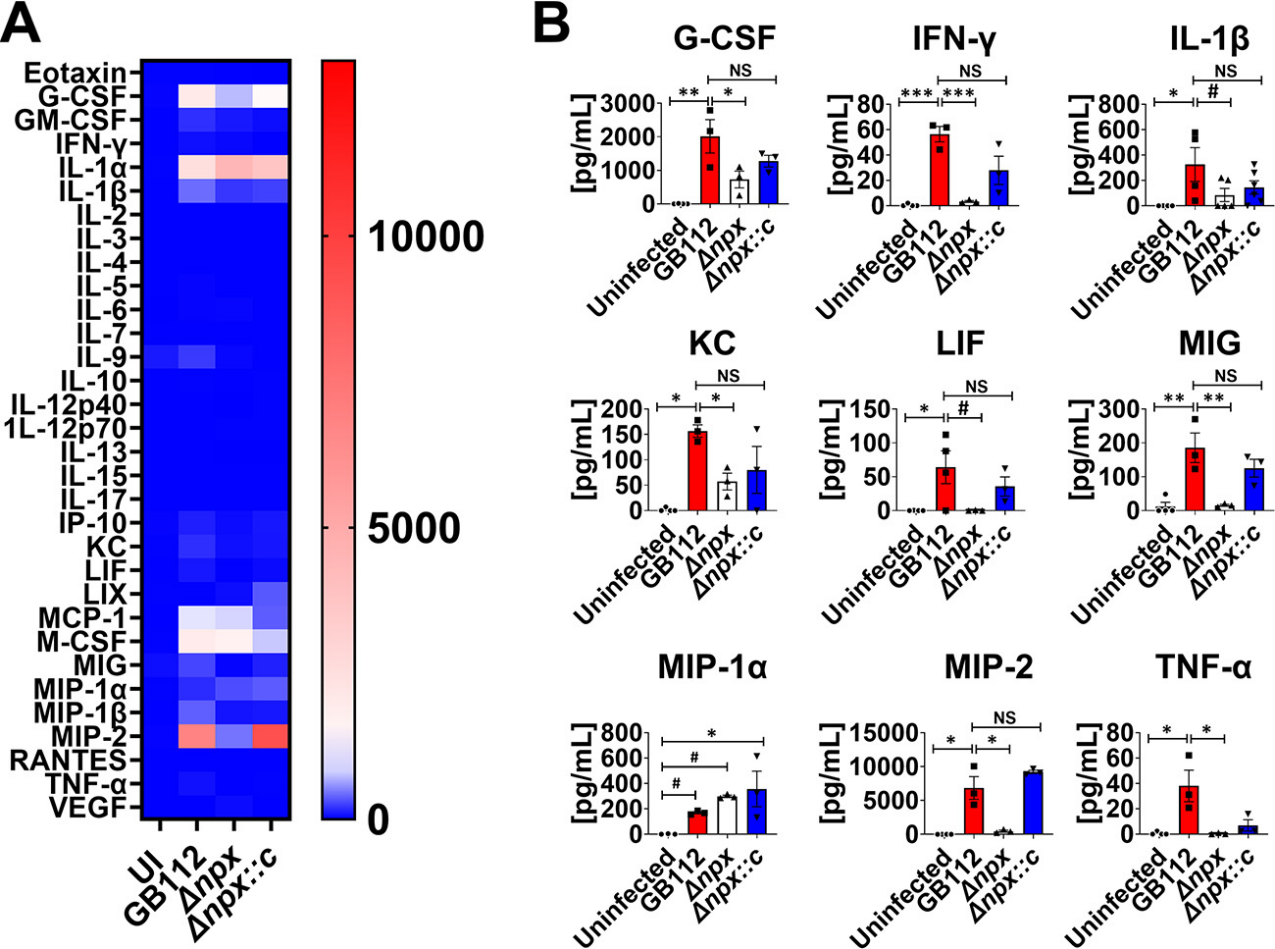
Analysis of cytokine production in amnion tissue in response to GBS infection. (A) Heat map results (blue, low levels; red, high levels) of multiplex cytokine analyses of amnion tissues after ascending vaginal infection with wild-type GB112, the Δ*npx* isogenic mutant, or the isogenic complemented Δ*npx* derivative (Δ*npx::c*) as well as the uninfected controls (UI). Amnion tissues were collected from pregnant mice on embryonic day 15.5, 2 days after vaginal infection with GBS. (B) Quantification of G-CSF, IFN-γ, IL-1β, KC, LIF, MIG, MIP-1α, MIP-2, and TNF-α levels revealed that wild-type GB112 infection (red bars) significantly enhances the production of these cytokines compared to the levels in the uninfected controls (black bars), but the isogenic Δ*npx* mutant (white bars) is significantly attenuated in its ability to induce these cytokines compared to the parental strain, except for MIP-1α. Conversely, the complemented derivative (Δ*npx::c*) (blue bars) is often statistically indistinguishable from the parental strain (not statistically significant [NS]). Bars indicate mean values ± standard errors of the means, with individual data points representing results from amnion tissues from individual fetal-placental units from separate dams. #, *P* < 0.05 (by Student’s *t* test). *, *P* < 0.05; **, *P* < 0.01; ***, *P* < 0.001; ****, *P* < 0.0001 (by one-way ANOVA with Tukey’s *post hoc* multiple-comparison test). The results indicate that GBS *npx* is required for the initiation of numerous proinflammatory cytokine responses in amnion tissues in a model of ascending infection during pregnancy.

**FIG 9:**
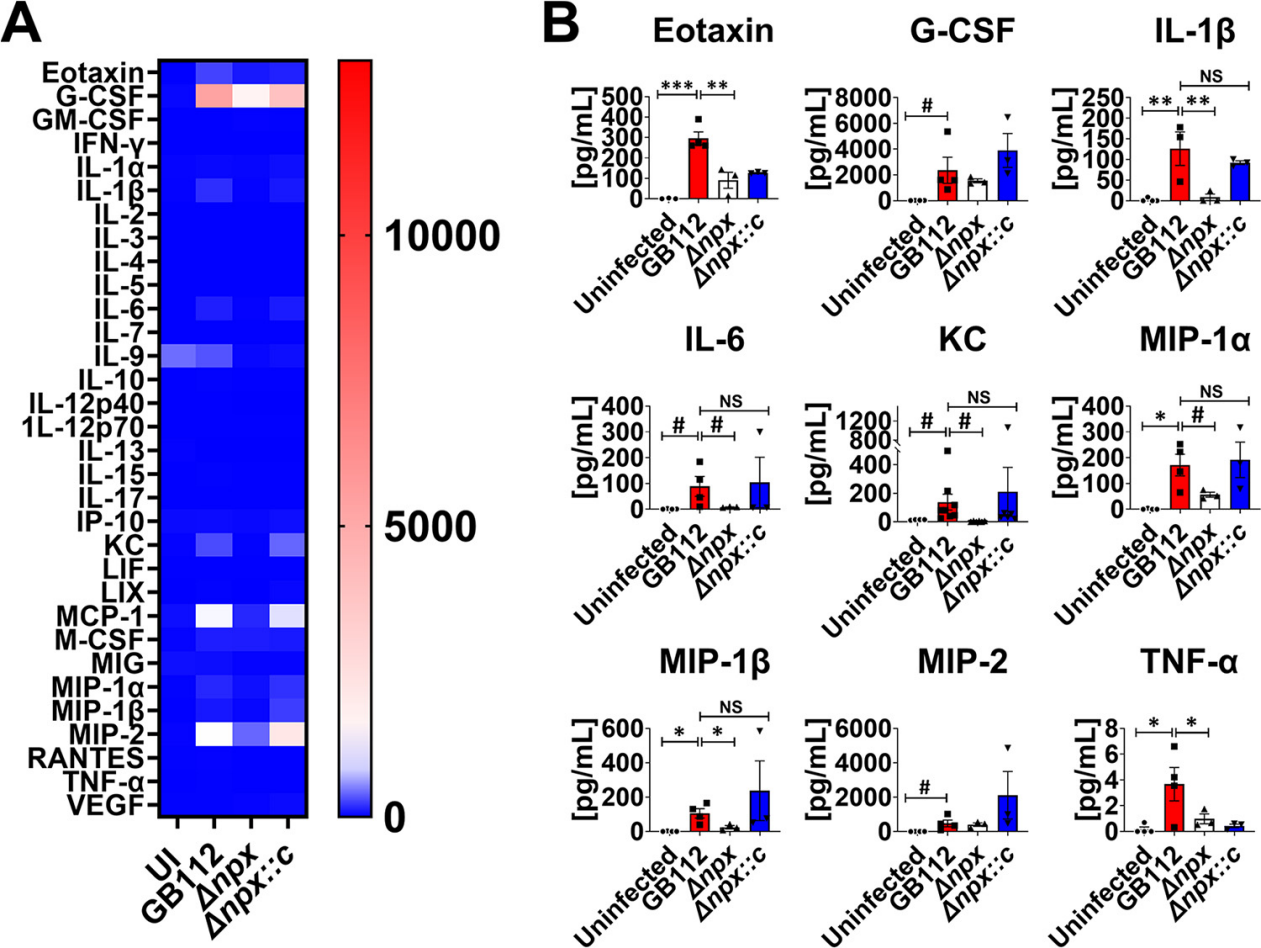
Analysis of cytokine production in fetus tissue in response to GBS infection. (A) Heat map results (blue, low levels; red, high levels) of multiplex cytokine analyses of fetal tissues after ascending vaginal infection with wild-type GB112, the Δ*npx* isogenic mutant, or the isogenic complemented Δ*npx* derivative (Δ*npx::c*) as well as the uninfected controls (UI). Fetus tissues were collected from pregnant mice on embryonic day 15.5, 2 days after vaginal infection with GBS. (B) Quantification of eotaxin, G-CSF, IL-1β, IL-6, KC, MIP-1α, MIP-1β, MIP-2, and TNF-α levels revealed that wild-type GB112 infection (red bars) significantly enhances the production of these cytokines compared to the levels in the uninfected controls (black bars), but the isogenic Δ*npx* mutant (white bars) is significantly attenuated in its ability to induce eotaxin, IL-1β, IL-6, KC, MIP-1α, MIP-1β, and TNF-α compared to the parental strain. Conversely, the complemented derivative (Δ*npx::c*) (blue bars) is statistically indistinguishable from the parental strain (not statistically significant [NS]) in its ability to induce IL-1β, IL-6, KC, MIP-1α, and MIP-1β. Bars indicate mean values ± standard errors of the means, with individual data points representing results from fetal tissues from individual fetuses from separate dams. #, *P* < 0.05 (by Student’s *t* test). *, *P* < 0.05; **, *P* < 0.01; ***, *P* < 0.001; ****, *P* < 0.0001 (by one-way ANOVA with Tukey’s *post hoc* multiple-comparison test). The results indicate that GBS *npx* is required for the initiation of numerous proinflammatory cytokine responses in fetal tissues in a model of ascending infection during pregnancy.

Because the Δ*npx* mutant showed decreased bacterial burdens and disease progression in the pregnant mice, we hypothesized that these could be attributed to changes in proinflammatory cytokine production as a consequence of bacterial infection. To test this, we utilized multiplex cytokine assays to quantify the repertoire of cytokines and chemokines produced within reproductive tissues in response to GBS infection and compared them to those produced in uninfected animal tissues. Our results indicate that infection with WT GBS significantly enhanced the production of IL-1β, MIP-1α, and TNF-α in the uterus, decidua, placenta, amnion, and fetus (Fig. 5 to 9 and Fig. S2 to S6) compared to the uninfected controls (*, *P* < 0.05 by one-way ANOVA; #, *P* < 0.05 by Student’s *t* test). Importantly, the deletion of the *npx* gene resulted in the significantly reduced production of these cytokines and chemokines compared to the levels in WT-infected samples (*, *P* < 0.05 by one-way ANOVA; #, *P* < 0.05 by Student’s *t* test). Similarly, granulocyte colony-stimulating factor (G-CSF), MCP-1, MIG, and MIP-2 were significantly upregulated in the uterus, decidua, placenta, and amnion of animals infected with WT GBS compared to the uninfected animals (*, *P* < 0.05 by one-way ANOVA; #, *P* < 0.05 by Student’s *t* test), and the loss of the *npx* gene resulted in significant reductions in the production of these cytokines and chemokines compared to the levels in WT-infected samples (*, *P* < 0.05 by one-way ANOVA; #, *P* < 0.05 by Student’s *t* test). IL-6 and MIP-1 were upregulated in the uterus, decidua, placenta, and fetus in response to WT GBS infection compared to the uninfected controls, and the deletion of the *npx* gene revealed significant reductions in the production of these cytokines and chemokines compared to the levels in WT-infected samples (*, *P* < 0.05 by one-way ANOVA; #, *P* < 0.05 by Student’s *t* test). IP-10 was upregulated in the uterus, decidua, and placenta in response to WT GBS infection compared to the uninfected controls, and significant reductions in the production of these cytokines and chemokines were observed with the inactivation of the *npx* gene compared to the levels in WT-infected samples (*, *P* < 0.05 by one-way ANOVA; #, *P* < 0.05 by Student’s *t* test). Complementation with the WT *npx* allele in *trans* restored cytokine and chemokine production to levels that were similar to those observed in WT GBS-infected animals or significantly higher than those measured in the isogenic Δ*npx* mutant-infected samples (*, *P* < 0.05 by one-way ANOVA; #, *P* < 0.05 by Student’s *t* test; NS, not statistically significant [statistically indistinguishable from WT GBS]).

10.1128/mbio.02870-22.2 FIG S2 Analysis of cytokine production in uterus tissue in response to GBS infection. Shown are the results of multiplex cytokine analyses of uterine tissues after ascending vaginal infection with wild-type GB112 (red bars), the Δ*npx* isogenic mutant (white bars), or the isogenic complemented Δ*npx* derivative (Δ*npx::c*) (blue bars) as well as the uninfected controls (UI) (black bars). Uterine tissues were collected from pregnant mice on embryonic day 15.5, 2 days after vaginal infection with GBS. Graphs indicate quantifications of IFN-γ, IL-2, IL-3, IL-4, IL-5, IL-7, IL-9, IL-10, IL-12p40, IL-12p70, IL-13, IL-15, IL-17, M-CSF, RANTES, and VEGF levels. Bars indicate mean values ± standard errors of the means, with individual data points representing results from uterus tissues from individual dams. *, *P* < 0.05 (by one-way ANOVA with Tukey’s *post hoc* multiple-comparison test). Download FIG S2, TIF file, 0.8 MB. Copyright © 2022 Lu et al. 2022 Lu et al. https://creativecommons.org/licenses/by/4.0/ This content is distributed under the terms of the Creative Commons Attribution 4.0 International license.

Infection with WT GBS resulted in the significantly enhanced production of select cytokines and chemokines, including eotaxin, G-CSF, granulocyte-macrophage colony-stimulating factor (GM-CSF), IL-1α, IL-1β, IL-6, IL-15, IP-10, KC, LIF, LIX, MCP-1, MIG, MIP-1α, MIP-1β, MIP-2, and TNF-α in the uterus (Fig. 5 and Fig. S2); eotaxin, granulocyte colony stimulating factor (G-CSF), granulocyte macrophage colony stimulating factor (GM-CSF), IL-1α, IL-1β, IL-6, IL-15, IP-10, KC, LIF, LIX, MCP-1, MIG, MIP-1α, MIP-1β, MIP-2, and TNF-α in the uterus (Fig. 5 and Fig. S2); eotaxin, G-CSF, GM-CSF, macrophage colony-stimulating factor (M-CSF), IL-1α, IL-1β, IL-6, interleukin 15 (IL-15), interferon gamma induced protein 10 (IP-10), keratinocyte chemoattractant (KC), leukemia inhibitory factor (LIF), lipopolysachharide-induced CXC chemokine (LIX), Monocyte chemoattractant protein-1 (MCP-1), Monokine induced by gamma (MIG), MIP-1α, MIP-1β, MIP-2, and TNF-α in the decidua (Fig. 6 and Fig. S3); G-CSF, IL-1β, IL-6, IP-10, KC, MCP-1, MIG, MIP-1α, MIP-1β, MIP-2, M-CSF, and TNF-α in the placenta (Fig. 7 and Fig. S4); G-CSF, M-CSF, IL-1β, KC, MCP-1, MIG, MIP-1α, MIP-2, and TNF-α in the amnion (Fig. 8 and Fig. S5); and eotaxin, IL-1β, IL-6, KC, MIP-1α, MIP-1β, and TNF-α in the fetus (Fig. 9 and Fig. S6), compared to the uninfected controls (*, *P* < 0.05 by one-way ANOVA; #, *P* < 0.05 by Student’s *t* test). The inactivation of the *npx* gene, however, evoked significant reductions in the production of many of these cytokines and chemokines compared to the levels in WT-infected samples (*, *P* < 0.05 by one-way ANOVA; #, *P* < 0.05 by Student’s *t* test).

10.1128/mbio.02870-22.3 FIG S3 Analysis of cytokine production in decidua tissue in response to GBS infection. Shown are the results of multiplex cytokine analyses of decidual tissues after ascending vaginal infection with wild-type GB112 (red bars), the Δ*npx* isogenic mutant (white bars), or the isogenic complemented Δ*npx* derivative (Δ*npx::c*) (blue bars) as well as the uninfected controls (UI) (black bars). Decidual tissues were collected from pregnant mice on embryonic day 15.5, 2 days after vaginal infection with GBS. Graphs indicate quantifications of IFN-γ, IL-2, IL-3, IL-4, IL-5, IL-7, IL-9, IL-10, IL-12p40, IL-12p70, IL-13, IL-15, IL-17, M-CSF, RANTES, and VEGF levels. Bars indicate mean values ± standard errors of the means, with individual data points representing results from decidual tissues from individual dams. *, *P* < 0.05; **, *P* < 0.01 (by one-way ANOVA with Tukey’s *post hoc* multiple-comparison test). Download FIG S3, TIF file, 0.9 MB. Copyright © 2022 Lu et al. 2022 Lu et al. https://creativecommons.org/licenses/by/4.0/ This content is distributed under the terms of the Creative Commons Attribution 4.0 International license.

10.1128/mbio.02870-22.4 FIG S4 Analysis of cytokine production in placenta tissue in response to GBS infection. Shown are the results of multiplex cytokine analyses of placental tissues after ascending vaginal infection with wild-type GB112 (red bars), the Δ*npx* isogenic mutant (white bars), or the isogenic complemented Δ*npx* derivative (Δ*npx::c*) (blue bars) as well as the uninfected controls (UI) (black bars). Placental tissues were collected from pregnant mice on embryonic day 15.5, 2 days after vaginal infection with GBS. Graphs indicate quantifications of eotaxin, GM-CSF, IFN-γ, IL-1α, IL-2, IL-3, IL-4, IL-5, IL-7, IL-9, IL-10, IL-12p40, IL-12p70, IL-13, IL-15, IL-17, LIF, LIX, RANTES, and VEGF levels. Bars indicate mean values ± standard errors of the means, with individual data points representing results from decidual tissues from individual dams. Download FIG S4, TIF file, 0.7 MB. Copyright © 2022 Lu et al. 2022 Lu et al. https://creativecommons.org/licenses/by/4.0/ This content is distributed under the terms of the Creative Commons Attribution 4.0 International license.

10.1128/mbio.02870-22.5 FIG S5 Analysis of cytokine production in amnion tissue in response to GBS infection. Shown are the results of multiplex cytokine analyses of placental tissues after ascending vaginal infection with wild-type GB112 (red bars), the Δ*npx* isogenic mutant (white bars), or the isogenic complemented Δ*npx* derivative (Δ*npx::c*) (blue bars) as well as the uninfected controls (UI) (black bars). Amnion tissues were collected from pregnant mice on embryonic day 15.5, 2 days after vaginal infection with GBS. Graphs indicate quantifications of eotaxin, GM-CSF, IL-1α, IL-2, IL-3, IL-4, IL-5, IL-6, IL-7, IL-9, IL-10, IL-12p40, IL-12p70, IL-13, IL-15, IL-17, IP-10, LIX, MCP-1, M-CSF, MIP-1β, RANTES, and VEGF levels. Bars indicate mean values ± standard errors of the means, with individual data points representing results from amnion tissues from individual dams. *, *P* < 0.05; **, *P* < 0.01; ***, *P* < 0.001 (by one-way ANOVA with Tukey’s *post hoc* multiple-comparison test). Download FIG S5, TIF file, 0.7 MB. Copyright © 2022 Lu et al. 2022 Lu et al. https://creativecommons.org/licenses/by/4.0/ This content is distributed under the terms of the Creative Commons Attribution 4.0 International license.

10.1128/mbio.02870-22.6 FIG S6 Analysis of cytokine production in fetus tissue in response to GBS infection. Shown are the results of multiplex cytokine analyses of fetal tissues after ascending vaginal infection with wild-type GB112 (red bars), the Δ*npx* isogenic mutant (white bars), or the isogenic complemented Δ*npx* derivative (Δ*npx::c*) (blue bars) as well as the uninfected controls (UI) (black bars). Fetal tissues were collected from pregnant mice on embryonic day 15.5, 2 days after vaginal infection with GBS. Graphs indicate quantifications of GM-CSF, IFN-γ, IL-1α, IL-2, IL-3, IL-4, IL-5, IL-7, IL-9, IL-10, IL-12p40, IL-12p70, IL-13, IL-15, IL-17, IP-10, LIF, LIX, MCP-1, M-CSF, MIG, RANTES, and VEGF levels. Bars indicate mean values ± standard errors of the means, with individual data points representing results from fetal tissues from individual dams. Download FIG S6, TIF file, 0.6 MB. Copyright © 2022 Lu et al. 2022 Lu et al. https://creativecommons.org/licenses/by/4.0/ This content is distributed under the terms of the Creative Commons Attribution 4.0 International license.

## DISCUSSION

While GBS has been identified as a perinatal pathogen since the 1930s, there are still major gaps in the knowledge of the pathophysiology of infection and disease outcomes by this bacterium. We previously observed that GBS interacts with gestational tissue macrophages and that GBS can invade the reproductive tract in a mouse model of ascending vaginal infection during pregnancy (17, 29, 38). We sought to understand the importance of individual GBS virulence factors that influence the outcome of GBS-macrophage interactions and might also have significance in clinical outcomes during pregnancy. We previously identified that a peroxide-detoxifying enzyme NADH peroxidase gene, *npx*, was upregulated when GBS was cultured with THP-1 macrophages (36). We further expanded that work in our current study by demonstrating that GBS *npx* aids in GBS survival within primary human placental macrophages.

Macrophages represent the second most common leukocytes within fetal membrane tissues, and these cells perform many roles, including regulating tissue remodeling during development and modulating maternal-fetal tolerance (39). Less is understood about the roles that macrophages may play during infection, as these cells are typically thought to be polarized to an anti-inflammatory M2 tolerogenic state (28). Some recent studies have noted that in response to bacteria, these cells change their polarization toward a more inflammatory M1 phenotype (40). GBS has evolved mechanisms such as a capsule to evade phagocytosis by placental macrophages (37), but once engulfed by innate immune cells, GBS deploys enhanced expression of the *npx* locus as a strategy to survive the peroxide stress encountered within the phagosomes of THP-1 macrophages (36).

Macrophages are implicated as a replicative niche for a variety of bacteria, including Pseudomonas aeruginosa (41), Yersinia pestis (42), Brucella neotomae (43), Escherichia coli (44), Neisseria gonorrhoeae (45), and Legionella pneumophila (46). Recent work has demonstrated that S. pneumoniae can survive and replicate within splenic macrophages, which serve as a reservoir for septicemia (47), and that group A Streptococcus can survive and replicate within human macrophages (48). These results mirror what we observed with GBS in primary human PMs.

Furthermore, macrophages have been implicated as a potential Trojan horse aiding in the dissemination of a variety of microbial pathogens, including Candida albicans (49), Mycobacterium tuberculosis (50), Toxoplasma gondii (51), Staphylococcus aureus (52), Cryptococcus neoformans (53), and Chlamydia trachomatis (54). The depletion of host macrophages impedes Chlamydia and GBS dissemination in the reproductive tract (38, 55), demonstrating the important role that intracellular bacterial survival within macrophages plays in bacterial invasion of the reproductive tract. Our previous results indicate that GBS utilizes *cadD*, a metal resistance determinant, to circumnavigate metal stress within placental macrophages and to enhance bacterial ascension and invasion of the gravid reproductive tract (38). Similarly, our current work demonstrates that GBS utilizes *npx*, a peroxide resistance determinant, to circumnavigate peroxide stress within placental macrophages and to aid in ascending infection and disease progression during pregnancy. Taken together, these results support the hypothesis that GBS could exploit macrophages as a Trojan horse to aid in the promotion of invasive bacterial infections during pregnancy.

Upon the recognition of pathogens by immune pattern recognition receptors (PRRs) such as Toll-like receptors, a cascade of responses by the macrophage is initiated. One mechanism of defense is the phagocytosis of the bacterial cell and chemical assault within the phagosome via the deployment of peroxides and reactive oxygen species (56). Highly reactive oxygen species can damage macromolecules, including lipids, proteins, and nucleic acids, ultimately leading to cell death (30). Within the reproductive tract, invading pathogens, including C. trachomatis (57) and N. gonorrhoeae (58), are assaulted with reactive oxygen species. There is an association between Chlamydia and spontaneous abortion, with oxidative stress being implicated, highlighting the oxidative response in the reproductive tract against invading pathogens (57). Additionally, the presence of ROS has been demonstrated in human amniotic fluid collected in the second and third trimesters of gestation (59). This likely presents an environmental challenge for bacteria, which are highly sensitive to oxidative stress, results that are supported by the survival and growth defects observed in the Δ*npx* mutant compared to the wild type and the complemented derivative grown in human or mouse amniotic fluid.

In response to this, bacterial pathogens have evolved a range of mechanisms to overcome ROS stress inside macrophages. For instance, S. aureus (60), P. aeruginosa (61, 62), Klebsiella pneumoniae (63), and M. tuberculosis (64) all express catalase to resist oxidative killing by macrophages. Other bacterial pathogens such as E. coli (65), Salmonella enterica serovar Typhi (66), and Burkholderia pseudomallei (67) express superoxide dismutase for the same purpose. GBS is catalase negative but expresses superoxide dismutase (SodA) (33). Our study demonstrates that the full repertoire of antioxidant defenses is required for invasive infection of the gravid reproductive tract and that the *npx-*encoded NADH peroxidase aids in GBS survival within reproductive tissue macrophages and is critical for full virulence in a pregnant animal model. Interestingly, a dye-neutralizing peroxidase (DyP) has been identified in M. tuberculosis, which is critical for bacterial survival within host macrophages as well (68). Similarly, in Listeria monocytogenes, peroxidases encoded within the *fri* and *ahpA* loci were each required for L. monocytogenes to survive acute peroxide stress, and the *fri* locus was essential for cytosolic growth within host macrophages (69). These studies further underscore the critical role that bacterial peroxidases play in the host-pathogen dialogue, specifically with respect to intracellular survival.

**FIG 10:**
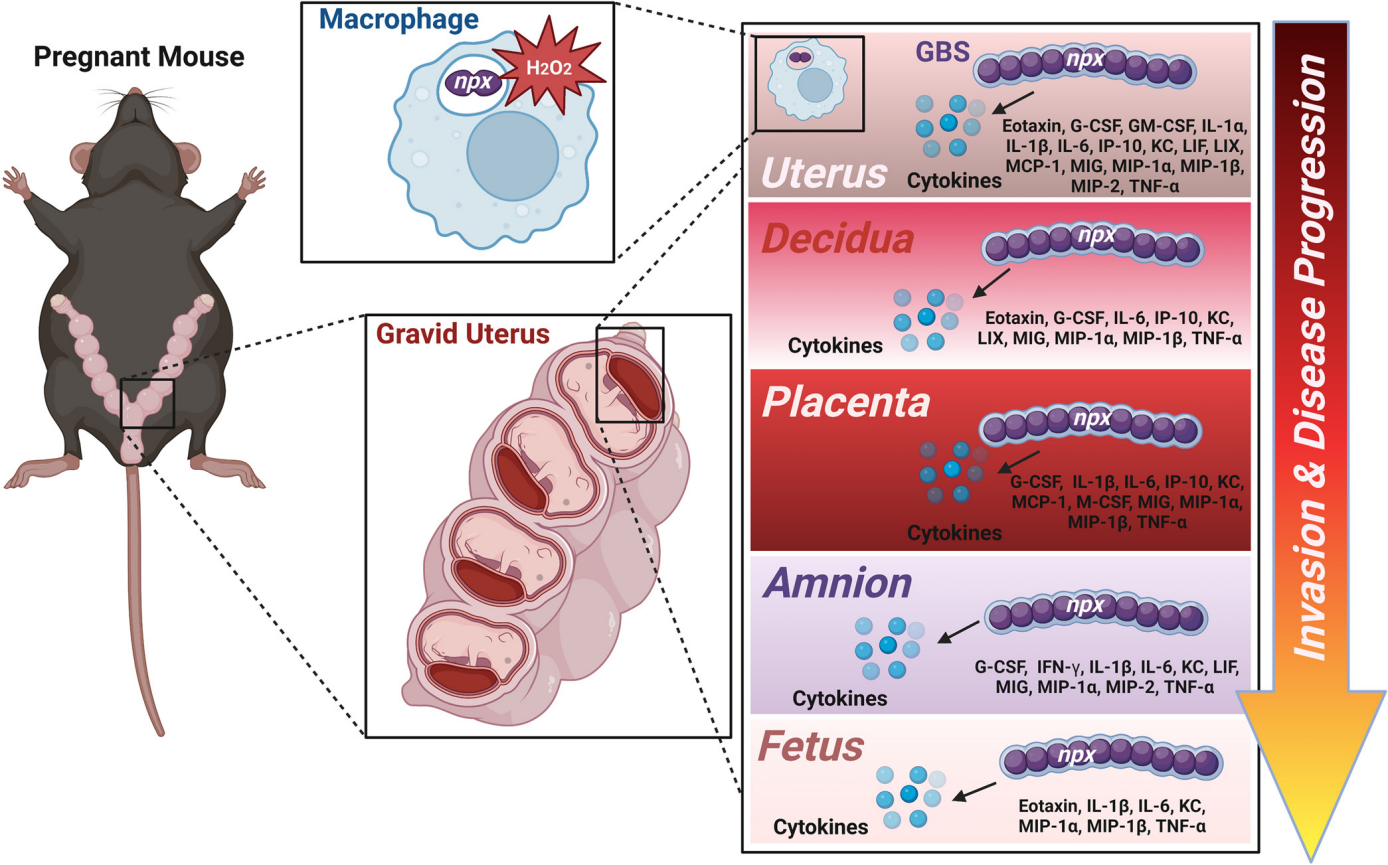
Conceptual model of *npx*-dependent invasion and proinflammatory signal initiation in ascending GBS infections during pregnancy. GBS ascends the reproductive tract by surviving within placental macrophages, in part by encoding an NADH peroxidase to aid in detoxifying oxidative stress (such as peroxides [H_2_O_2_]). GBS invasion and full burdens within the gravid reproductive tract (uterus, decidua, placenta, amnion, and fetus) require *npx* expression and trigger the production of tissue compartment-specific proinflammatory cytokines, resulting in inflammation and disease progression, including rupture of membranes, preterm birth, and maternal demise. (Image created with BioRender.com.)

In addition to aiding in GBS intracellular survival within host immune cells, the *npx* locus is critical for virulence *in vivo*. In our mouse model of ascending vaginal infection during pregnancy, we observed that mutants lacking *npx* showed impaired invasion of gestational tissues, cognate inflammatory responses, and disease compared to the parental strain or the complemented mutant, demonstrating the importance of *npx* for pathogenesis (Fig. 10). We observed drastic reductions in the bacterial burdens in the uterus, decidua, placenta, amnion, and fetal tissue compartments derived from animals infected with the Δ*npx* mutant compared to the burdens in animals infected with the parental strain or the complemented isogenic derivative. These reductions in burdens correlated with the decreased production of proinflammatory cytokines such as IL-1β, MIP-1α, and TNF-α in all of the examined tissue compartments of the gravid reproductive tract. Interestingly, the diminutions of proinflammatory cytokine production and bacterial burdens were associated with cognate decreases in adverse pregnancy outcomes such as PPROM, preterm birth, and maternal demise. Previous work has linked high levels of IL-1β, MIP-1α, and TNF-α with an enhanced risk of preterm birth (70). It is likely that the expression of proinflammatory cytokines perturbs maternal tolerance of the semiallogeneic fetus, leading to an enhanced risk of adverse pregnancy outcomes (71). Recently, interest has been piqued in exploiting the NLRP3 inflammasome pathway as a potential chemotherapeutic strategy to ameliorate the risk associated with perinatal disease outcomes (72). Because the expression of the *npx* locus is critical for GBS ascension of the reproductive tract and the initiation of these signaling pathways that promote inflammation, a dually targeted approach of the inhibition of the GBS NADH peroxidase and the NLRP3 inflammasome pathway could prove useful in combating GBS perinatal infections.

Intracellular infection of PMs resulted in the production of proinflammatory cytokines such as G-CSF, GM-CSF, growth-regulated protein alpha (GRO-α), IL-1RA, IL-1α, IL-1β, IL-6, IL-8, MCP-1, MIP-1α, MIP-1β, and TNF-α. However, *npx* was dispensable for this induction. This was a surprising result considering the significant differences in bacterial loads within these macrophages, underscoring that even low levels of GBS intracellular infection are sufficient to induce the production of these cytokines. The production of proinflammatory cytokines by macrophages instigates the recruitment of neutrophils in mice upon GBS infection (15, 73). A similar response has also been observed in humans (73). Neutrophils aid in the clearance of bacteria by phagocytosis and subsequent killing in internal vacuoles called phagosomes (74). Finally, neutrophils excrete neutrophil extracellular traps (NETs) loaded with various antimicrobial peptides (75). These extracellular DNA traps have been exhibited in response to GBS infection (15). Collectively, placental macrophages and neutrophils predominate the innate immune response against GBS infection.

Our studies indicate that NADH peroxidase plays an important role in the full virulence of GBS in a mouse model of ascending vaginal infection during pregnancy. Other reactive oxygen species-detoxifying enzymes such as catalase and superoxide dismutase are also important for the virulence of many bacterial pathogens, especially those that survive and establish replicative niches within macrophages (76, 77). The introduction of bacterium-specific inhibitors of oxidative stress response pathways may target bacteria without impacting the host, providing exciting new avenues for drug development. As such, developing small molecules or other chemotherapeutic strategies to inhibit these enzymes may be a viable option to defend against these bacterial infections. The identification of these bacterium-specific inhibitors is being researched currently and includes small molecules that inhibit M. tuberculosis catalase (78). A plausible future direction of our work may include screening preexisting banks of small molecules against purified GBS NADH peroxidase protein or creating a crystal structure to identify regions for targeted drug design.

## MATERIALS AND METHODS

### Bacterial strains and culture conditions.

S. agalactiae strain GB00112 (GB112), which represents the wild-type (WT) or parental strain, was utilized in this study. GB112 is a clinical, sequence type 12 (ST-12), capsular polysaccharide (CPS) serotype III strain isolated from a rectovaginal swab of a postpartum patient (79). We previously examined GB112 for interactions with host macrophages and fetal membranes (36, 80). Isogenic GB112 mutants were constructed previously. These mutants include an *npx* deletion mutant (Δ*npx*) and a complemented Δ*npx* mutant harboring a plasmid containing the *npx* locus (Δ*npx::c*), as previously described (36). Bacterial strains were grown on tryptic soy agar plates supplemented with 5% sheep blood or in Todd-Hewitt broth (THB) at 37°C. Derivatives harboring the pLZ12 plasmid were grown in medium supplemented with 3 μg/mL chloramphenicol. E. coli DH5α strains used for the mutation and complementation processes were grown in LB broth or agar supplemented with either 150 μg/mL erythromycin or 20 μg/mL chloramphenicol when necessary.

### Purification of placental macrophages.

Deidentified placental tissue was collected from nonlaboring women who delivered healthy, full-term infants by Caesarian section at the Vanderbilt University Medical Center with approval from the Vanderbilt University Medical Center Institutional Review Board (VUMC IRB) (approval number 181998). Placental macrophages (PMs) were isolated according to our previously described methods (80, 81). Briefly, villous core tissue was macerated and enzymatically digested with hyaluronidase, collagenase, and DNase (Sigma-Aldrich) before being strained through a stainless steel filter and suspended in RPMI 1640 with HEPES, l-glutamine, and fetal bovine serum supplemented with antibiotic and antifungal factors. Cells were filtered and centrifuged, and CD14^+^ cells were isolated using the magnetic activated cell sorting (MACS) cell separation system with CD14 microbeads (Miltenyi Biotec). Cells were incubated in RPMI 1640 medium (Thermo Fisher) with 10% charcoal-stripped fetal bovine serum (FBS) (Thermo Fisher) and a 1% antibiotic-antimycotic solution (Thermo Fisher) overnight at 37°C in 5% carbon dioxide. The following day, PMs were suspended in RPMI 1640 medium without antibiotic-antimycotic and distributed into polystyrene plates. Cells were seeded at a density of 200,000 cells per well in a polystyrene, 24-well culture plate in RPMI 1640 with a 1% antibiotic-antimycotic solution and 10% charcoal dextran FBS (RPMI^+/+^) and then incubated for 24 h in a humidified atmosphere at 37°C with 5% CO_2_.

### Coculture of GBS with placental macrophages and survival assays.

PMs were cultured in RPMI 1640 medium (Thermo Fisher) with 10% charcoal-stripped fetal bovine serum (Thermo Fisher) and a 1% antibiotic-antimycotic solution (Thermo Fisher) overnight at 37°C in room air supplemented with 5% carbon dioxide. Cocultured cells were incubated at 37°C in air supplemented with 5% carbon dioxide for 1 to 24 h with medium free of the antibiotic-antimycotic solution. Macrophages were inoculated at a multiplicity of infection (MOI) of 10:1 bacteria to host cells for 1 h. Cocultures were washed with sterile medium, resuspended in fresh medium containing 100 μg/mL of gentamicin (Sigma) to kill extracellular bacteria, and further incubated for 4 h at 37°C. Gentamicin kills extracellularly located GBS but is limited in its ability to gain access to intracellular organisms. Subsequently, the samples were extensively washed with sterile phosphate-buffered saline (PBS) and dislodged with trypsin (1× 0.05% trypsin-EDTA; Gibco). Following the collection of the PMs, the cells were lysed by the addition of 1 mL of distilled water (dH_2_O). Cellular cytoplasmic contents were serially diluted in PBS and plated onto blood agar plates to determine the number of viable intracellular bacteria. Samples containing only bacteria were used to estimate the efficacy of antibiotic killing as a control experiment (82).

### Transmission electron microscopy analyses.

Cocultures of GBS and macrophages were subjected to primary fixation with 2.5% glutaraldehyde and 2.0% paraformaldehyde in 0.05 M sodium cacodylate buffer at room temperature for 24 h. Subsequently, samples were washed three times with 0.05 M sodium cacodylate buffer and subjected to a secondary fixation step with 0.1% osmium tetroxide for 15 min. Samples were washed three times with 0.05 M sodium cacodylate buffer before being sequentially dehydrated with increasing concentrations of ethanol. After dehydration, samples were embedded in resin, polymerized, and sectioned into 70- to 90-nm sections via ultramicrotomy. Sections were lifted onto nickel or copper 100-mesh grids (Electron Microscopy Sciences) and secondarily stained with 1% phosphotungstic acid. Grids were imaged with a Philips/FEI T12 transmission electron microscope to visualize intracellular bacteria. Bacteria were enumerated in a blind manner using the ImageJ software package.

### Ascending vaginal infection model.

GBS infection of pregnant mice and subsequent analyses were performed as previously described (15, 37). Briefly, C57BL/6J mice were purchased from Jackson Laboratories and mated in harem breeding strategies (1 male to 3 to 4 females) overnight. Pregnancy was confirmed by the presence of a vaginal mucus plug establishing the embryonic date (embryonic day 0.5 [E0.5]) the following day. On E13.5, pregnant dams were anesthetized via the inhalation of isoflurane and vaginally infected with 5 × 10^2^ to 10^4^ CFU in 0.05 mL of THB plus 10% gelatin. Uninfected controls were also maintained. Infections were allowed to progress until term (E21.5) for disease progression studies or until E15.5 for bacterial and immunological assays. Animals were euthanized by carbon dioxide asphyxiation, and necropsy was performed to harvest reproductive tissues, including the uterus, placenta, decidua, amnion, and fetus tissues.

### Quantitative culture evaluation of bacterial burdens.

To determine the bacterial burden in reproductive tissues, quantitative culture methods were employed, as previously described (37). Briefly, reproductive tissues were weighed and placed into sterile THB. Tissues were homogenized, subjected to serial dilution, and plated onto blood agar to quantify bacteria (CFU per milligram) in host tissue. Plates were incubated at 37°C in air supplemented with 5% carbon dioxide overnight, and individual colonies were counted to quantify CFU.

### Evaluation of bacterial growth in amniotic fluid.

To determine bacterial growth in human or mouse amniotic fluid, quantitative culture methods were employed, as described above. Briefly, human amniotic fluid was pipetted off the surface of the placentas from term, nonlaboring C-section deliveries. Mouse amniotic fluid was collected after necropsy on embryonic day 15.5. All amniotic fluid samples were stored at −80°C until utilization for growth assays. Amniotic fluid samples (100 L) were placed into 96-well plates, and a 1:100 dilution of a bacterial culture grown overnight (either WT GBS, the Δ*npx* mutant, or the complemented derivative) was inoculated into the amniotic fluid. Samples were incubated overnight (16 to 24 h) at 37°C in room air supplemented with 5% CO_2_. The following day, viable bacteria were enumerated via serial dilution and plating onto bacteriological medium (blood agar plates). Plates were incubated at 37°C in room air supplemented with 5% CO_2_ overnight, and the following day, bacterial colonies were counted to enumerate viable bacterial cells.

### Evaluation of cytokine responses to GBS infection.

Mouse reproductive tissues, maternal sera, and amniotic fluid were analyzed by multiplex cytokine assays. Mouse tissues were placed into 0.5 mL of sterile PBS or THB plus 10 mg/mL penicillin, homogenized, and passed through a 0.22-μm filter. Samples were frozen at −80°C or on dry ice until analyses were performed. Samples were analyzed by Eve Technologies (Alberta, Canada) via a multiplex cytokine array, as previously described (83). Validation of host targets for specific cytokines (IL-1β, IL-6, KC, and TNF-α) was performed by a sandwich enzyme-linked immunosorbent assay (ELISA) (Abcam), as indicated in our previous study (84).

### Ethics statement.

This study was carried out in accordance with the recommendations of the Vanderbilt University Medical Center Institutional Review Board. This protocol was approved by the IRB (approval numbers 181998 and 00005756). All animal experiments were performed in accordance with the Animal Welfare Act, U.S. federal law, and NIH guidelines. All experiments were carried out under a protocol approved by the Vanderbilt University Institutional Animal Care and Use Committee (IACUC) (approvals M/14/034, M/17/002, and V2000089), a body that has been accredited by the Association for Assessment and Accreditation of Laboratory Animal Care (AAALAC).

### Statistical analyses.

Statistical analysis of parametric data with more than two groups was performed using one-way ANOVA with either Tukey’s or Dunnett’s *post hoc* correction for multiple comparisons; all reported *P* values were adjusted to account for multiple comparisons. For parametric data with two groups, Student’s *t* test or one-way ANOVA was used. *P* values of ≤0.05 were considered significant. Nonparametric data (such as log-transformed CFU data) were analyzed by Mann-Whitney U or Kruskal-Wallis tests. Disease outcome data, including survival curves, were analyzed using the Mantel-Cox log rank test and the Gehan-Breslow-Wilcoxon test. All data analyzed in this work were derived from at least three biological replicates (representing different human or mouse samples). Statistical analyses were performed using GraphPad Prism 9 (GraphPad Software Inc.).

### Data availability.

Data are available upon reasonable request to the authors.
