# Supplementary figures and images for: Streptococcus agalactiae npx Is Required for Survival in Human Placental Macrophages and Full Virulence in a Model of Ascending Vaginal Infection during Pregnancy
Source: mBio. 2022 Nov 21;13(6):e02870-22. doi: 10.1128/mbio.02870-22 (PMC9765263; doi:10.1128/mbio.02870-22)

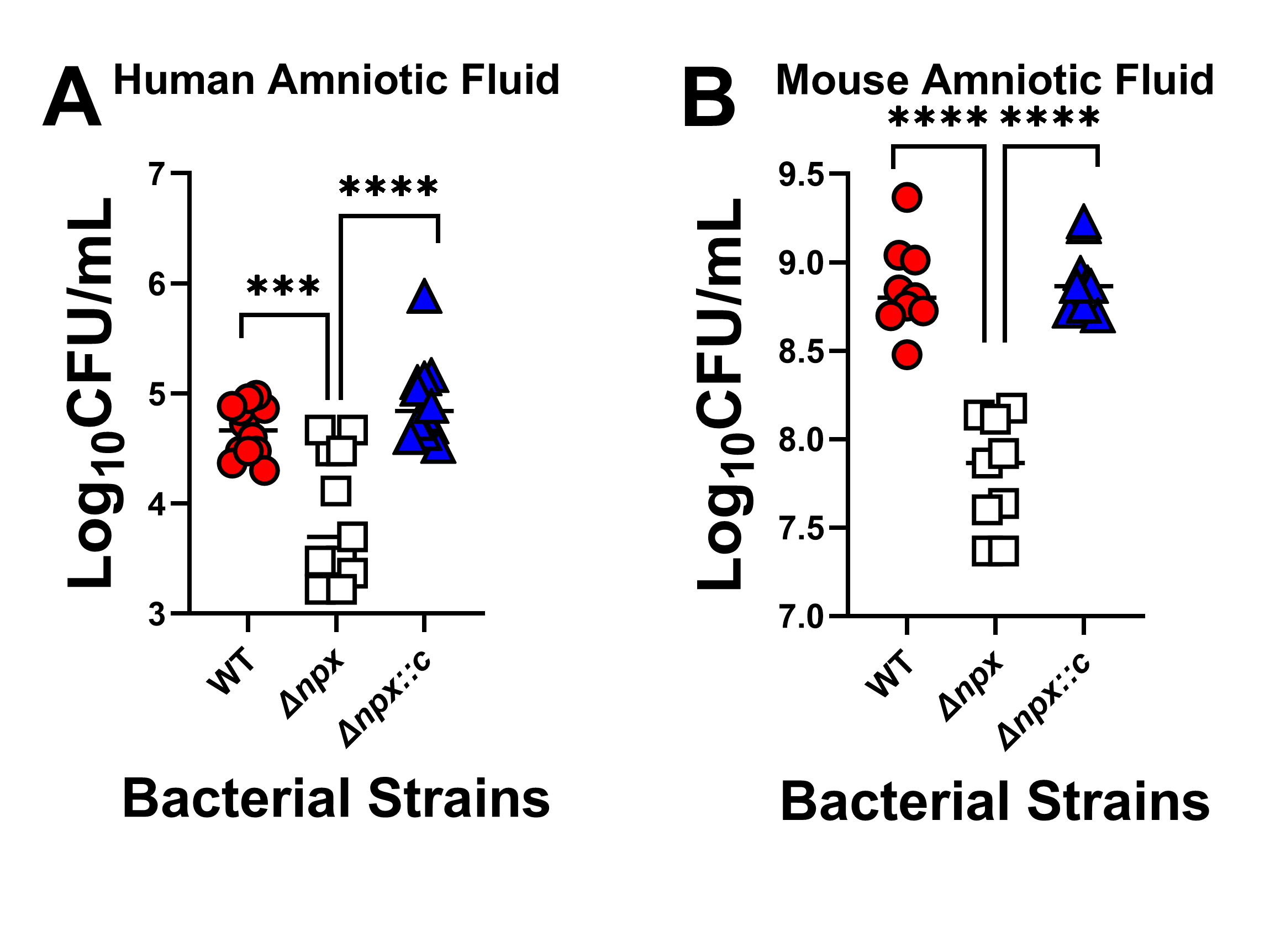

Supplement: FIG S1 [file mbio.02870-22-s0001.tif]

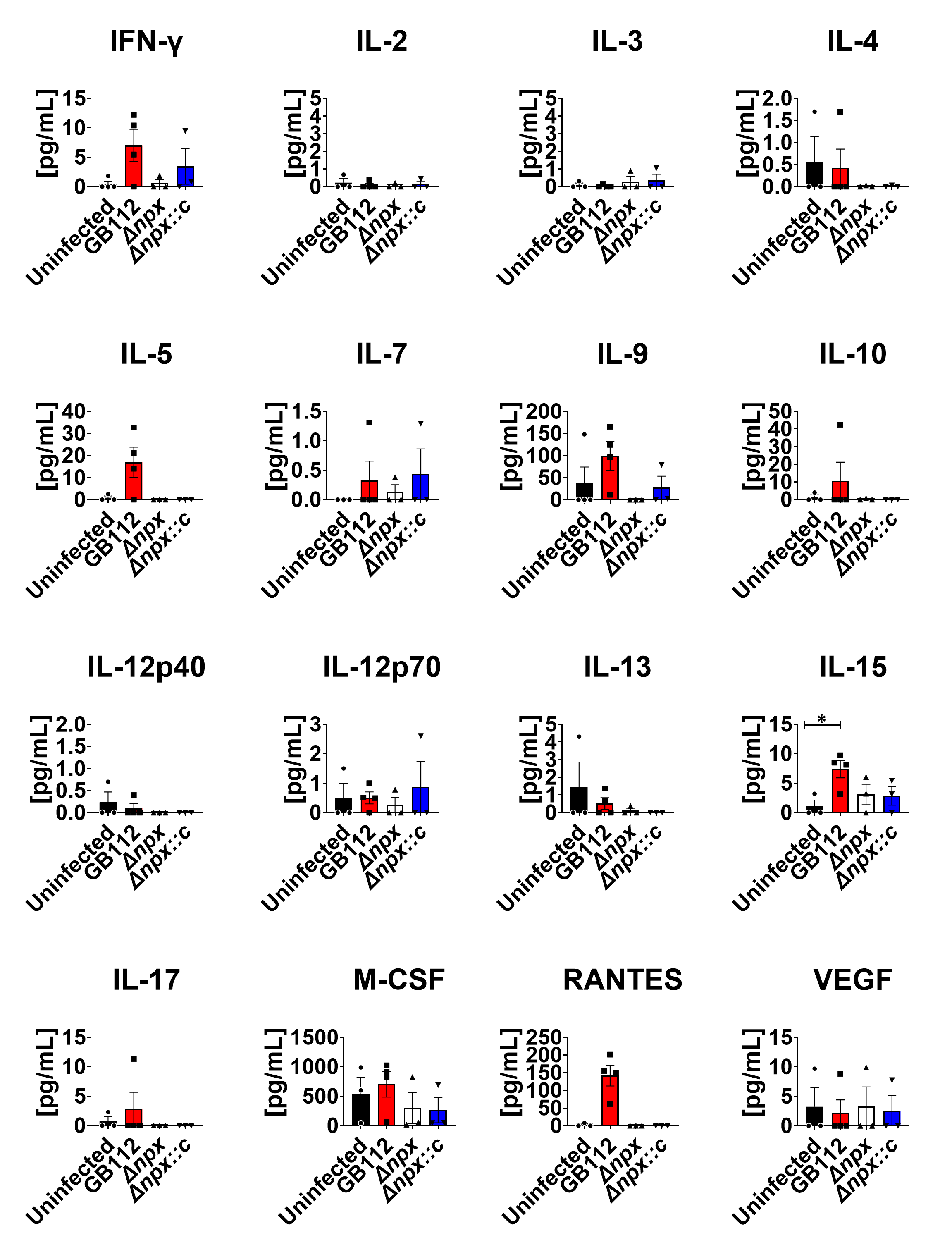

Supplement: FIG S2 [file mbio.02870-22-s0002.tif]

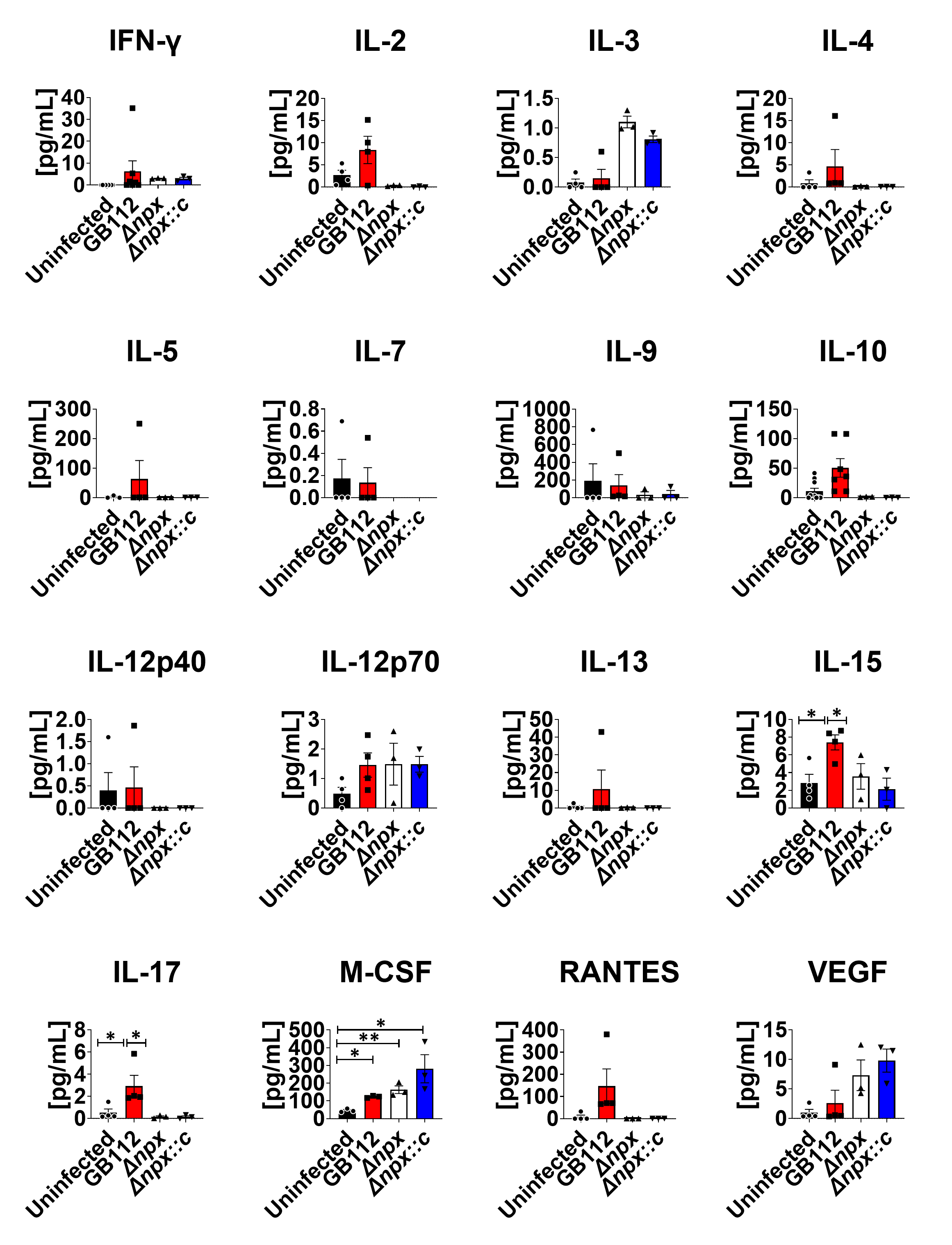

Supplement: FIG S3 [file mbio.02870-22-s0003.tif]

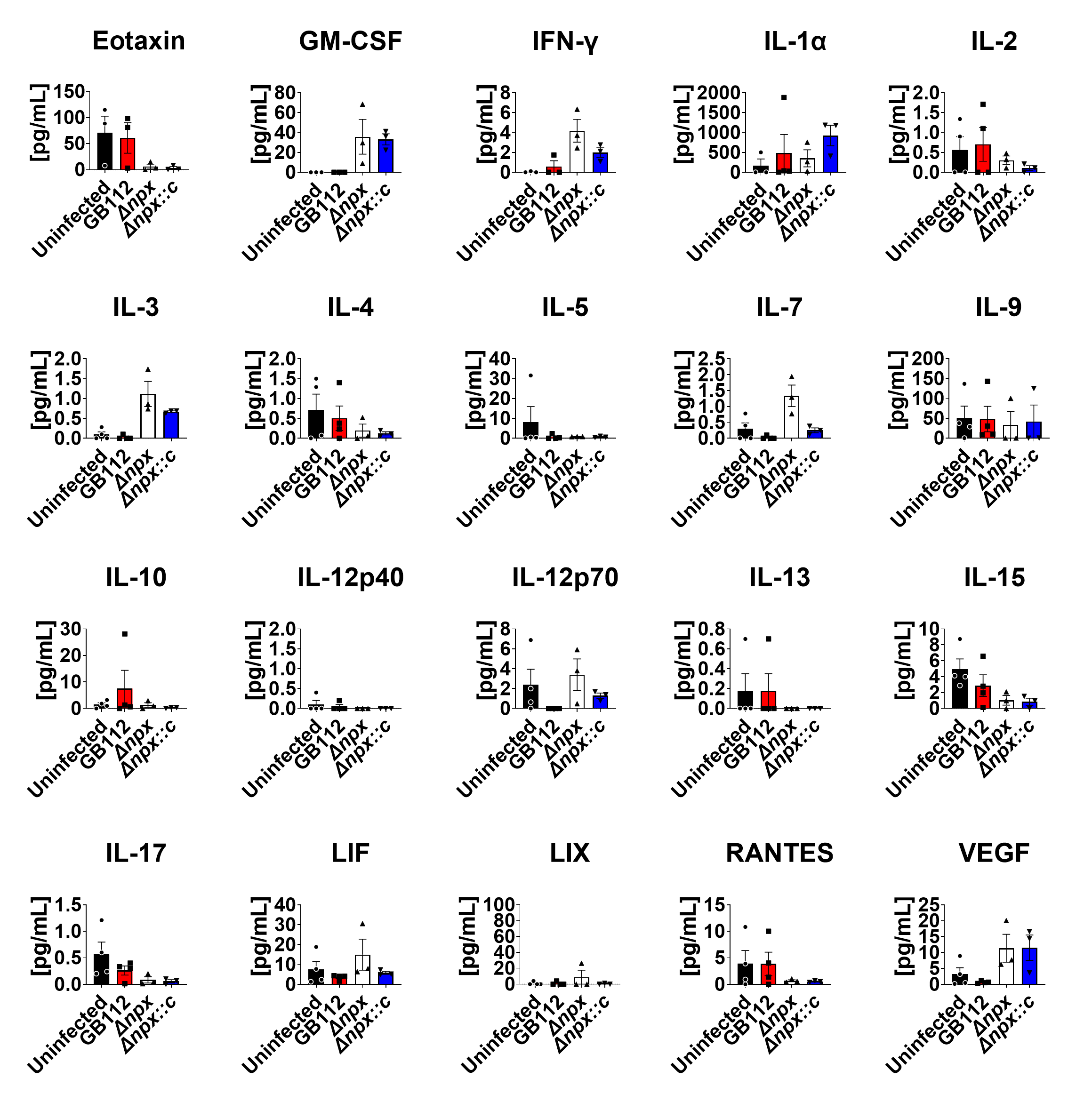

Supplement: FIG S4 [file mbio.02870-22-s0004.tif]

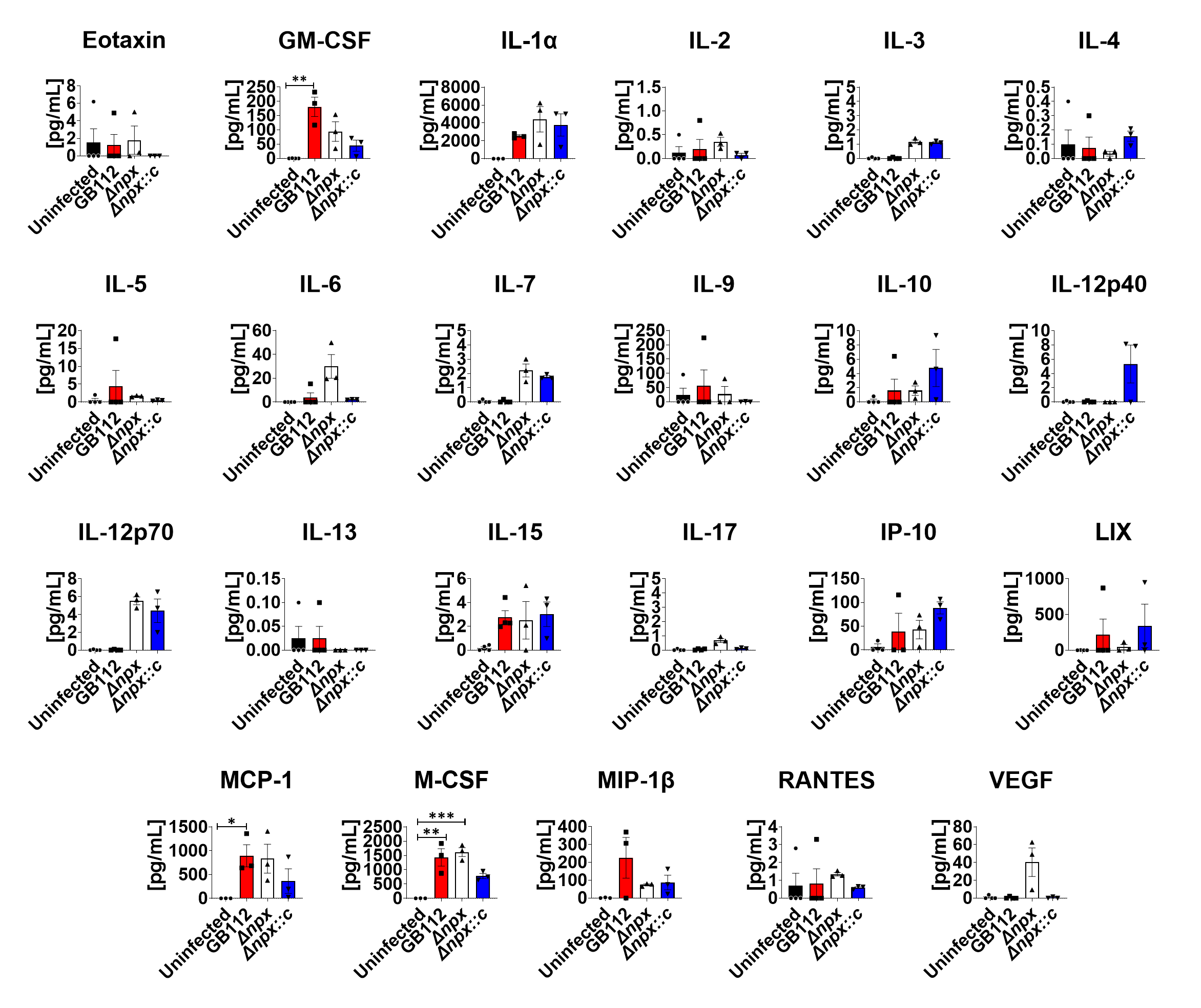

Supplement: FIG S5 [file mbio.02870-22-s0005.tif]

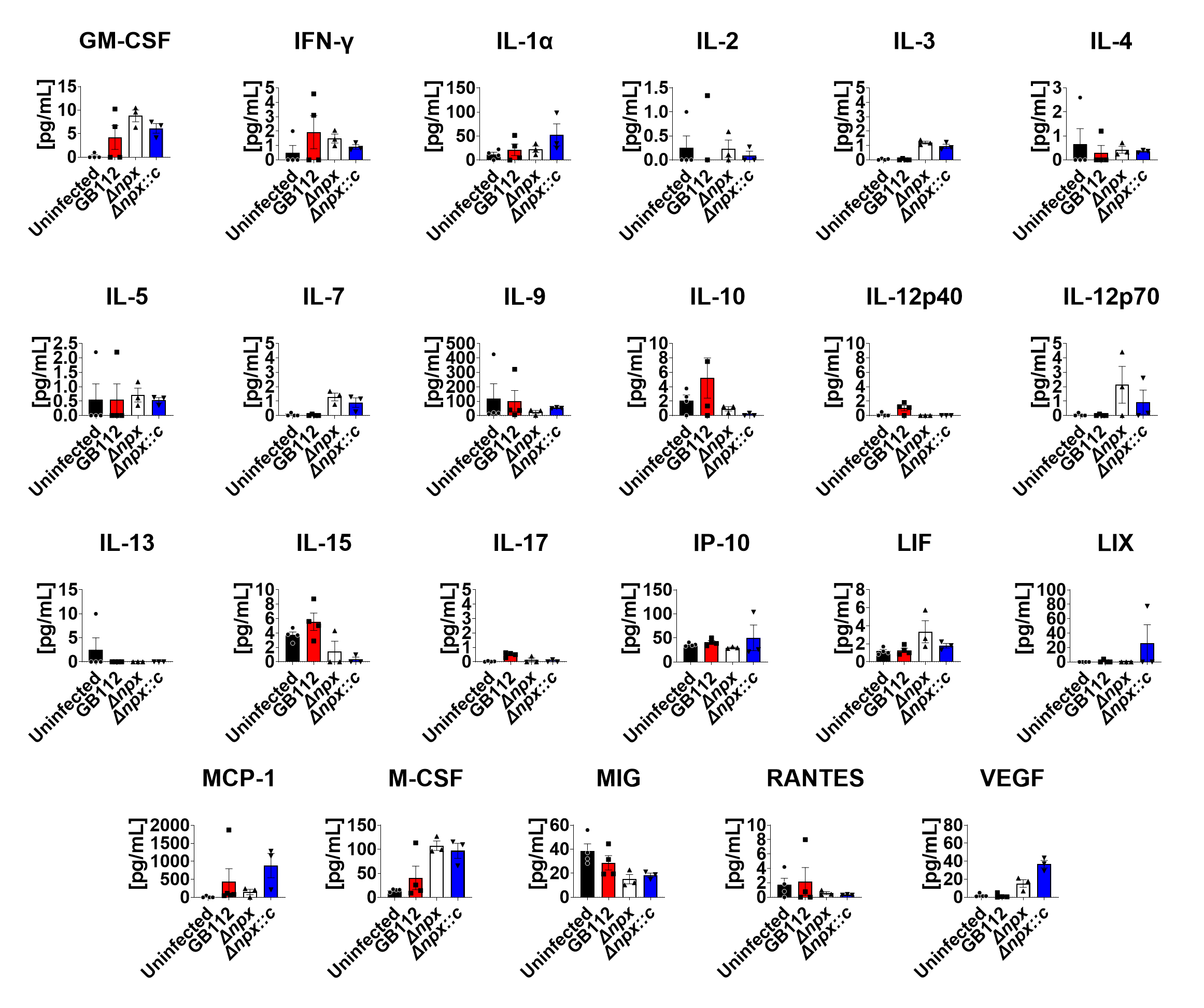

Supplement: FIG S6 [file mbio.02870-22-s0006.tif]
